# Microbial community composition and metabolic potential during a succession of algal blooms from *Skeletonema* sp. to *Phaeocystis* sp.

**DOI:** 10.3389/fmicb.2023.1147187

**Published:** 2023-04-17

**Authors:** Jianming Zhu, Si Tang, Keke Cheng, Zhonghua Cai, Guofu Chen, Jin Zhou

**Affiliations:** ^1^School of Marine Science and Technology, Harbin Institute of Technology, Weihai, Shandong, China; ^2^Shenzhen Public Platform for Screening and Application of Marine Microbial Resources, Institute for Ocean Engineering, Shenzhen International Graduate School, Tsinghua University, Shenzhen, Guangdong, China

**Keywords:** algal bloom, succession, microbial communities and functions, *Skeletonema* sp., *Phaeocystis* sp.

## Abstract

Elucidating the interactions between algal and microbial communities is essential for understanding the dynamic mechanisms regulating algal blooms in the marine environment. Shifts in bacterial communities when a single species dominates algal blooms have been extensively investigated. However, bacterioplankton community dynamics during bloom succession when one algal species shift to another is still poorly understood. In this study, we used metagenomic analysis to investigate the bacterial community composition and function during algal bloom succession from *Skeletonema* sp. to *Phaeocystis* sp. The results revealed that bacterial community structure and function shifted with bloom succession. The dominant group in the *Skeletonema* bloom was *Alphaproteobacteria*, while *Bacteroidia* and *Gammaproteobacteria* dominated the *Phaeocystis* bloom. The most noticeable feature during the successions was the change from *Rhodobacteraceae* to *Flavobacteriaceae* in the bacterial communities. The Shannon diversity indices were significantly higher in the transitional phase of the two blooms. Metabolic reconstruction of the metagenome-assembled genomes (MAGs) showed that dominant bacteria exhibited some environmental adaptability in both blooms, capable of metabolizing the main organic compounds, and possibly providing inorganic sulfur to the host algae. Moreover, we identified specific metabolic capabilities of cofactor biosynthesis (e.g., B vitamins) in MAGs in the two algal blooms. In the *Skeletonema* bloom, *Rhodobacteraceae* family members might participate in synthesizing vitamin B_1_ and B_12_ to the host, whereas in the *Phaeocystis* bloom, *Flavobacteriaceae* was the potential contributor for synthesizing vitamin B_7_ to the host. In addition, signal communication (quorum sensing and indole-3-acetic acid molecules) might have also participated in the bacterial response to bloom succession. Bloom-associated microorganisms showed a noticeable response in composition and function to algal succession. The changes in bacterial community structure and function might be an internal driving factor for the bloom succession.

## 1. Introduction

Marine phytoplankton is a major contributor to primary production, performing more than half of the photosynthetic carbon fixation and playing an important role in regulating global climate (Keller and Riebesell, 1989). However, algal blooms result from the localized and transient rapid growth of phytoplankton species, causing an imbalance of ecological function (Grattan et al., 2016; Morabito et al., 2018). Algal blooms are occurring at increasing frequency and are considered harmful due to the toxins produced by the algae, which directly poison animals or indirectly threaten human health (Grattan et al., 2016; Morabito et al., 2018). Considerable efforts have been made to elucidate and discuss the causes of algal blooms from environmental, food safety, and biological perspectives (Zohdi and Abbaspour, 2019).

Similar to the plant rhizosphere, the phycosphere environment is important for algae-bacteria interactions (Seymour et al., 2017). In this micro-niche, complex interactions occur between phytoplankton and bacteria, including mutualisms, commensalisms, competition, and parasitism (Amin et al., 2012; Fuentes et al., 2016). Researchers have found that microorganisms significantly impact algal bloom formation and are more critical than environmental parameters (Needham and Fuhrman, 2016; Teeling et al., 2016). Recent studies have shown that the shift patterns of bacteria contribute to bloom formation and extinction (Cui et al., 2020; Shao et al., 2020; Zhang et al., 2020). However, the current understanding of the response of bacterial communities to bloom development is inadequate and only addresses the different phases of a single species in the bloom process (Zhou et al., 2020a,b).

In natural marine environments, phytoplankton blooms are usually caused by multiple phytoplankton species. It was previously reported that diatoms were coupled and often co-exist with *Noctiluca scintillans*, making it a mixed-species ecosystem in bloom area (Dwivedi et al., 2015). The coexistence of diatoms and *Phaeocystis pouchetii* during bloom process was previously reported (Larsen et al., 2004). It is important to emphasize that the dominant species often show succession during a bloom event (Dwivedi et al., 2015; Xu et al., 2020). In a typical succession of marine algal bloom, diatoms dominate initially, followed by smaller motile phytoplankton (Zhang et al., 2015; Xu et al., 2020). For example, in the eastern English Channel and the Southern Bight of the North Sea, phytoplankton spring blooms were characterized by a typical succession from diatom (i.e., *Skeletonema costatum*, *Brockmaniella brockmanii*, and *Ditylum brightwelli*) to *Phaeocystis* (i.e., *P. globosa*) (Schapira et al., 2008; Grattepanche et al., 2010, 2011). These different phytoplankton species have different physiological properties and secreted metabolites, which form contrasting nutritional regimes causing algal species-specific activation of bacterial communities and function (Amin et al., 2012; Sarmento et al., 2013; Livanou et al., 2017). However, limited information is available on the response of microbial communities to the dramatic shift in algal species’ succession during a mixed phytoplankton bloom. Therefore, studying the response of bacterial communities and functions to species succession during a mixed-species bloom event will improve our understanding of phytoplankton-bacterial interactions in the ocean. In the Shenzhen coastal area, annual bloom succession by two microalgae species (such as *Skeletonema* sp. to *Phaeocystis* sp.) is frequent and provides an opportunity to study the characteristics of microorganisms associated to algal bloom succession events (Liu et al., 2022). Therefore, the environmental samples here allowed us to study the characteristics of microorganisms in the process of algal bloom succession.

Community-level gene analysis has provided critical functional insights into the bacteria during a bloom event; however, it could not link function with phylogeny or individual populations (Zhou et al., 2020b). High-throughput sequencing-based approaches contribute fundamentally to understand aquatic ecosystems by informing us how ecosystem functions are distributed across time, space, and taxa (Sunagawa et al., 2015). Metagenomics provides extensive inventories of community metabolic and functional capabilities combined with genome binning algorithms, thus linking ecosystem processes to specific microbial taxa (Quince et al., 2017).

Here, we have designed an artificial simulation experiment using plankton samples from the Shenzhen coastal waters and successfully induced an algal bloom in the laboratory, where a discernable succession phenomenon from *Skeletonema* sp. to *Phaeocystis* sp. algae was observed. Furthermore, we conducted metagenomic binning to obtain an extensive catalog of microbial genomes sampled across the succession process. We hypothesize that microbial change is a potential driving force for algal bloom species succession, which involves the changes in bacterial community structure and function. Based on this assumption, we aimed to (1) investigate the composition and succession of bacterioplankton communities associated with two different algal blooms; (2) identify potential core bacterial taxa that could be considered biomarkers for different algal bloom processes; (3) reveal the mechanism of algae-bacteria interactions during the bloom process and how the bacteria influence bloom development; and (4) illuminate the regulation mechanisms of algal bloom succession from a microbial perspective (bacterial composition and function) and describe algae-bacteria interactions and roles in the mixed algal bloom process.

## 2. Materials and methods

### 2.1. Experimental setup

An artificial algal bloom induction experiment was performed from 28 October 2020 (day 0, sample G0) to 26 November 2020 (day 30, samples G1 to G29). The microcosm photobioreactor (PBR) (100 l) was designed according to previous methods (Wu et al., 2021). A total of six PBRs were used, and each acted as an independent biological replicate. Before the experiment, all the PBRs were filled with natural seawater from Dapeng Bay, South China Sea (22°32’N, 114°22′E). To simulate a natural algal bloom, we induced phytoplankton growth by supplementing external nutrients on the first day. The initially added nutrients in the microcosms were NaNO_3_ (8.83 × 10^−4^ M), NaH_2_PO_4_·2H_2_O (3.63 × 10^−5^ M), Na_2_SiO_3_·9H_2_O (1.07 × 10^−4^ M), and FeCl_3_·6H_2_O (1.16 × 10^−5^ M). During the experiment, all PBRs were exposed to artificial light at a surface irradiance of approximately 200 μE m^−2^ s^−1^ in a 12:12 h light–dark cycle, maintaining water temperature at 24 ± 1°C. For proper liquid circulation to avoid cell accumulation, each photobioreactor had an internal draught tube to achieve the flow pattern of an airlift loop. For gas injection, a ceramic diffuser was placed at the bottom of the photobioreactor to ensure sufficient gas supply. Air was continuously injected into the PBR at 0.1 l min^−1^ and a flow meter was installed to maintain the flow rate.

### 2.2. Algal bloom monitoring and samples collection

Algal biomass was monitored daily by sampling. Fifty-milliliter samples were collected daily from the PBRs to determine algal biomass. Part of the samples was fixed with 1% Lugol iodine for algal identification and quantification. The algal cells were observed and counted using a hemocytometer under an optical microscope (magnification × 100) (Axiostar, Zeiss, Germany).

We divided the PBRs into three groups to obtain samples for metagenomic sequencing. Each sample collected from two PBRs was mixed as an independent biological replicate to satisfy the DNA extraction demands for metagenomic analysis. The sampling frequency was performed once every 2 days. The collected samples (2 l) were successively filtered through a 300-mesh plankton net to remove larger particles. Water samples were then filtered through a 3-μm fiber membrane (47 mm in diameter; code: FSLW, Millipore, USA) to collect the first subset of microorganisms (i.e., the attached taxa). The filtrate was then passed through a 0.22-μm fiber membrane (47 mm in diameter; code: GSWP, Millipore, USA) to obtain the second subset (i.e., the free-living taxa). The filters were then flash-frozen in liquid nitrogen and stored at −80°C for subsequent DNA extraction.

### 2.3. DNA extraction and metagenomic sequencing

Total DNA was extracted from filters using a FastDNA Spin Kit (mBio, USA) according to the manufacturer’s instructions. The concentration and purity of extracted DNA were determined with a TBS-380 Mini-Fluorometer (Turner Biosystems, Sunnyvale, CA, USA) and a NanoDrop2000 Spectrophotometer (Thermo Fisher Scientific, MA, USA), respectively. The DNA extract was fragmented to an average size of about 400 bp using Covaris M220 (Gene Company Limited, China) for paired-end library construction. Paired-end library was constructed using NEXTflex™ Rapid DNA-Seq (Bioo Scientific, Austin, TX, USA). Paired-end sequencing was performed on an Illumina Hiseq instrument (Illumina Inc., San Diego, CA, USA) at Majorbio Bio-Pharm Technology Co. (Shanghai, China) using HiSeq Reagent kits according to the manufacturer’s instructions.^1^ In total, 18 metagenomes (i.e., three biological replicates from six time points during algal succession) were sequenced.

### 2.4. Metagenome assembly, binning, and quality screening

The raw reads from metagenome sequencing were cleaned by removing adaptor sequences, trimming, and low-quality reads (reads with N bases, a minimum length threshold of 50 bp, and a minimum quality threshold of 20) using fastp (version 0.20.0) (Chen et al., 2018). The resulting high-quality reads were then assembled into contigs using MEGAHIT (version 1.1.2) (parameters: kmer_min = 47, kmer_max = 97, step = 10) (Li et al., 2015). Contigs with a length of 300 bp or more were selected as the final assembling results. Open reading frames (ORFs) in contigs were identified using MetaGene (Noguchi et al., 2006). A nonredundant gene catalog was constructed using CD-HIT (version 4.6.1) with 90% sequence identity and coverage (Fu et al., 2012). Following quality control, reads were mapped to the nonredundant gene catalog with 95% identity using SOAPaligner, and gene abundance in each sample was evaluated (Li et al., 2008). Representative sequences in the nonredundant gene catalog were annotated based on the KEGG (Kyoto Encyclopaedia of Genes and Genomes) database through blastp with an e-value cutoff of 1e^−5^ using Diamond (v0.9.19) for metabolic pathway annotations (Buchfink et al., 2015). To avoid bias, the abundance of specific genes was calculated by normalizing the reference sequence length and sequencing depth. The abundance of the genes was calculated using the following equation:


$$\mathrm{Abundance}\;\left(\mathrm{RPKM}\right)=\sum_{1}^{n}\frac{N_{\mathrm{mapped reads}}\times 150/L_{\mathrm{reference sequence}}}{N_{\mathrm{meta}}\times 10^{-6}}$$

where N_mapped reads_ is the number of the reads in the metagenome data that mapped to the reference sequence; L_reference sequence_ is the sequence length of the corresponding specific reference sequence; n is the number of reads belonging to the same subtype of the gene (e.g., KO); 150 is the sequence length of the Illumina reads; and N_meta_ is the number of reads in the metagenome sequence data (10 million).

Clean reads were then used to obtain metagenome-assembled genomes (MAGs) using the metaWRAP Binning module (−maxbin2 –concoct –metabat2 options) (Uritskiy et al., 2018). We consolidated the resulting bins into a final bin set with the metaWRAP Bin_refinement module (−c 70 -x 10 options) and the Reassemble_bins module. We quantified the bins (i.e., results from the Bin_refinement module) with Salmon using the Quant_bins module with default parameters (Patro et al., 2017). The relative abundance of each MAG from the separate sample was calculated with coverM v0.2.0.^2^ These decontaminated bins were then dereplicated at ANI ≥99% using dRep v2.6.2 (Olm et al., 2017).

### 2.5. Taxonomic assignment and functional annotation of MAGs

The taxonomy of the high-quality MAGs (bins) was classified using GTDB-Tk v1.3.0 (Chaumeil et al., 2019). MAGs were functionally annotated with Prokka (Seemann, 2014). Functional annotations were assigned with enrichM v0.4.7^3^ using the KEGG Orthology database (Kanehisa et al., 2015). Phylogenetic relationships among the MAGs were inferred by constructing a maximum-likelihood tree using bacterial marker genes identified in GTDB-Tk. The bacterial reference tree was inferred with IQ-Tree v1.6.9 (Nguyen et al., 2014). The tree was visualized and annotated in the interactive Tree of Life (iTol).^4^

### 2.6. Statistical analysis

The relative abundance of each MAG from the separate sample was used to estimate community composition. Bacterial community composition was visualized using principal coordinate analysis (PCoA) based on the Bray–Curtis dissimilarity metric. Permutational analysis of variance (PERMANOVA) was performed on community data using the vegan package (Dixon, 2003). The relative abundance of MAGs was z-score standardized for comparative analysis across different MAGs in the heat map. Potential statistically significant bacterial taxa in the various algal bloom processes were identified through the two-tailed t-test using STAMP (v.2.1.3) with adjusted *p* values (Parks et al., 2014). To detect bacterial KEGG pathways with significantly different abundances between bloom samples, linear discriminant analysis effect size (LEfSe) was used according to the online protocol.^5^ Alpha diversity of each sample was assessed by the Shannon’s diversity index. Statistical differences in alpha diversity among the different algal bloom stages were calculated using analysis of variance (ANOVA) at the *p* < 0.05 significance level. Data analyses were performed using the SPSS software package 13.0 (Armonk, NY, United States).

## 3. Results

### 3.1. Characteristics of the algal blooms

**Figure 1:**
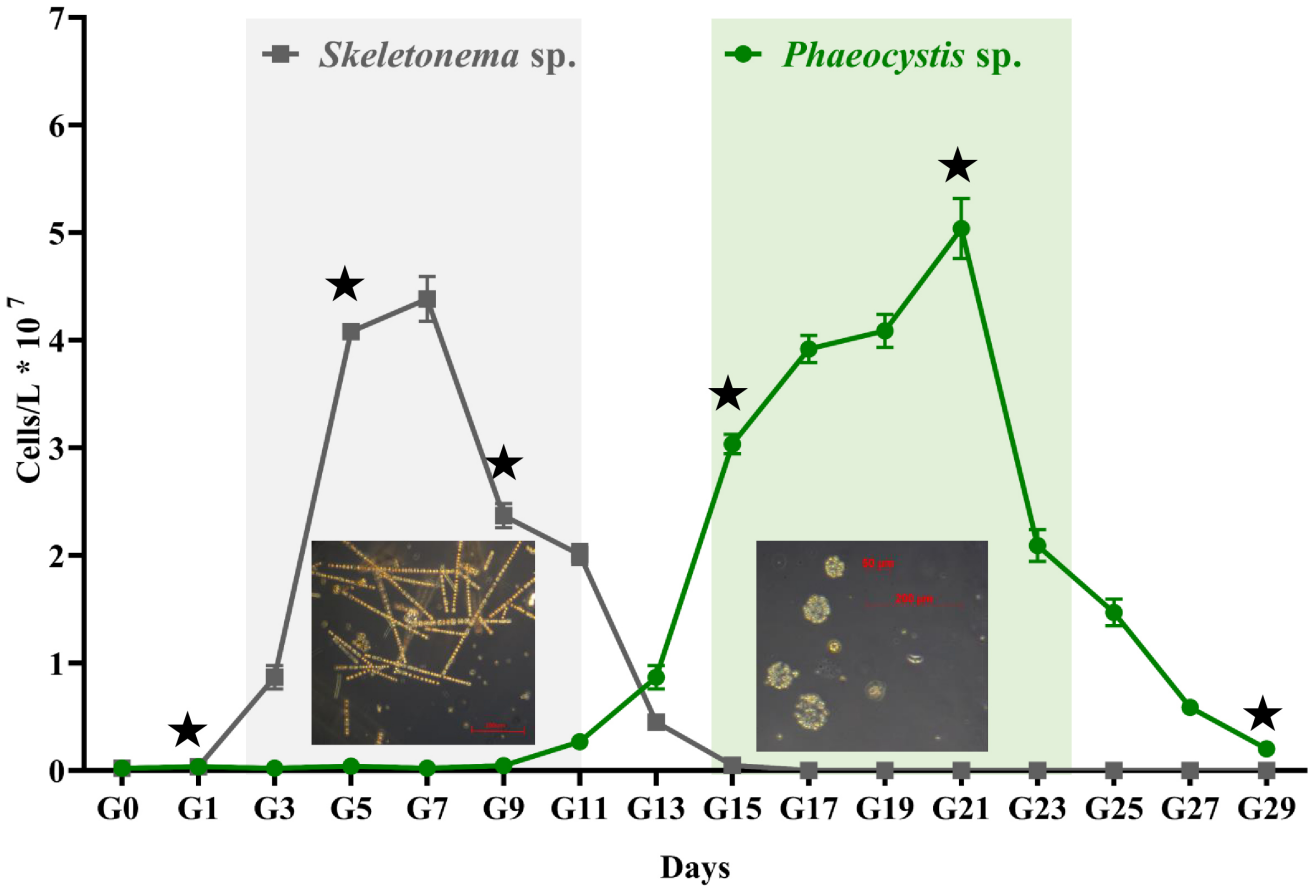
The abundance of algal cells counts across the experiment. The gray and green panels represent the *Skeletonema* sp. and *Phaeocystis* sp. blooms, respectively. Error bars indicate SD (*n* = 6). The asterisk denotes the sampling time for metagenomic sequencing analysis.

Figure 1 shows the progression for the artificially induced algal blooms. Sample G0 is the seawater from Dapeng Bay, and G1 to G29 represents the time points (days) in the algal bloom period. As seen in Figure 1, an algal bloom event was successfully induced. Three distinct algal bloom stages were observed, i.e., pre-stage (G1–G3), middle-stage (G3–G25), and post-stage (G25–G29). The bloom experienced one algal succession. The dominant species in the first (G3–G13) and the second half (G15–G25) of the middle-stage were *Skeletonema* sp. and *Phaeocystis* sp., respectively. For the former, the cells were 2–12 μm in diameter and formed long chains, each containing up to 60 cells with highly variable intercellular distances. For the latter, the solitary cells, either flagellated or non-flagellated, were generally 2–8 μm in diameter. More commonly, most solitary cells formation colonies, which are composed of embedded mucus-encrusted cells up to 0.8 cm in diameter. The cell density of *Skeletonema* sp. ranged from 1.0 × 10^6^ to 4.6 × 10^7^ cells/L, with the highest biomass appearing at G7. During the *Skeletonema* bloom, time points G1, G5, and G9 represented the beginning, developmental, and death stages, respectively. Points G13 and G15 signified the transitional period in which a mixture of various algae coexisted. From the time point G15, *Phaeocystis* sp. became the dominant species, with a maximum abundance at G21 (5.01 × 10^7^ cells/L). During the *Phaeocystis* bloom phase, points G15, G21, and G29 were the beginning, developmental, and death stages, respectively.

### 3.2. Genomic reconstruction and taxonomic identification of MAGs

**Figure 2:**
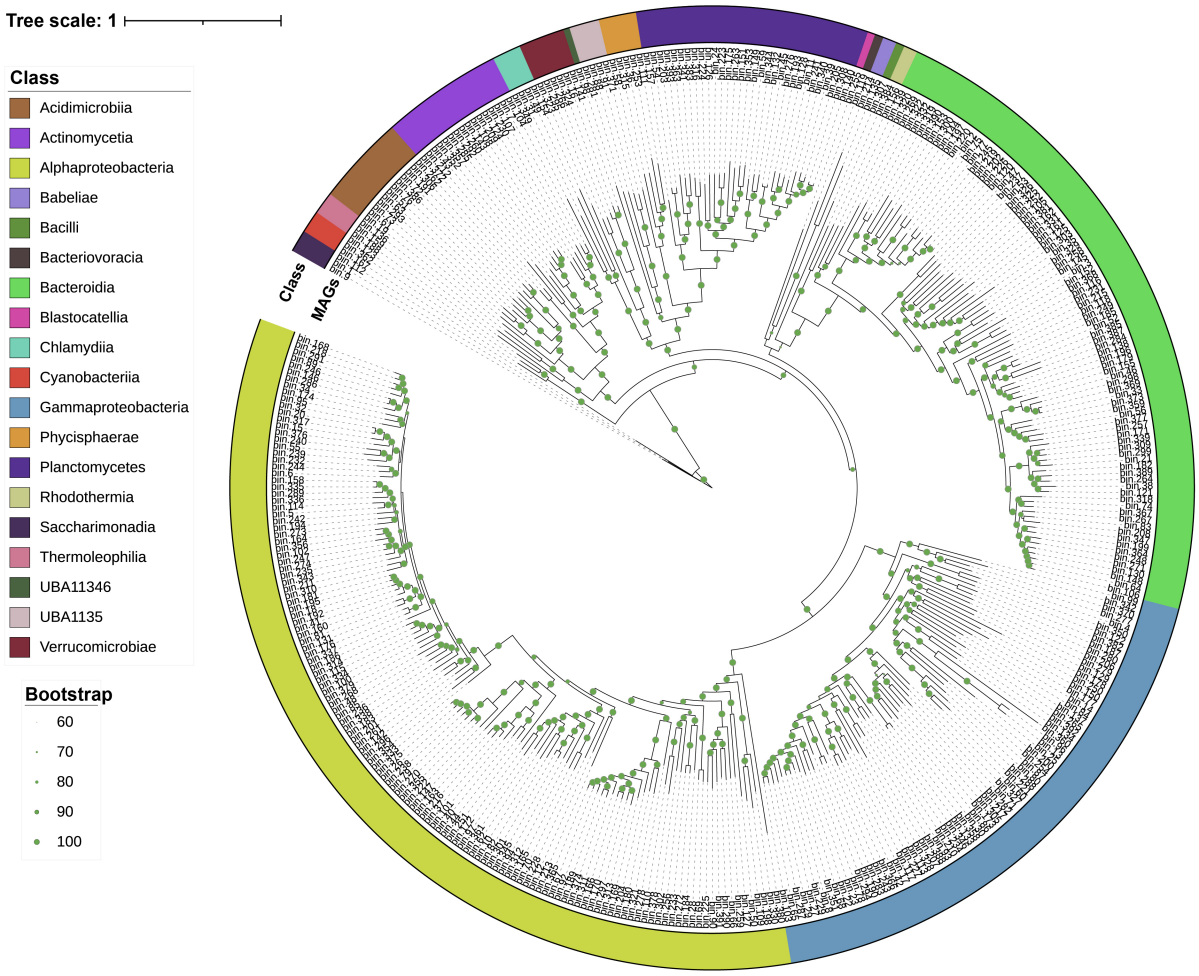
The maximum-likelihood tree based on conserved genes. Taxonomic classifications were determined using GTDB-tk. The external ring (colored) indicates the bacterial taxonomy (i.e., class information) to which the MAGs belong. Branch labels display the temporary name for the MAGs. Green dots represent branch support values between 60 and 100%.

Metagenomic assembly and binning were used for a genome-resolved analysis of *Skeletonema* sp. and *Phaeocystis* sp. blooms. In total, 389 bacterial MAGs were screened (completeness >70%, contamination <10%) for subsequent analysis (see Supplementary Table S1 for further taxonomic classification and relative abundance). The MAGs were further analyzed to identify their potential influence on the bloom processes. A phylogenetic reconstruction using conserved single-copy protein-coding genes provided an overview of the bacterial diversity associated with the blooms (Figure 2). At the phylum level, Proteobacteria dominated in the *Skeletonema* sp. bloom, while Bacteroidetes dominated in the *Phaeocystis* sp. bloom (Supplementary Figure S1). These bacterial MAGs belonged to 19 classes, including the *Alphaproteobacteria* (133 MAGs), *Bacteroidia* (88), *Gammaproteobacteria* (73), *Planctomycetes* (31), *Actinomycetia* (16), *Acidimicrobiia* (12), *Verrucomicrobiae* (6), *Phycisphaerae* (5), *Chlamydiia* (4), UBA1135 (4), *Saccharimonadia* (3), *Cyanobacteriia* (3), *Thermoleophilia* (3), *Candidatus* Babeliae (2), *Rhodothermia* (2), *Bacteriovoracia* (1), *Bacilli* (1), UBA11346 (1), and *Blastocatellia* (1).

### 3.3. Microbial community dynamics and diversity

**Figure 3:**
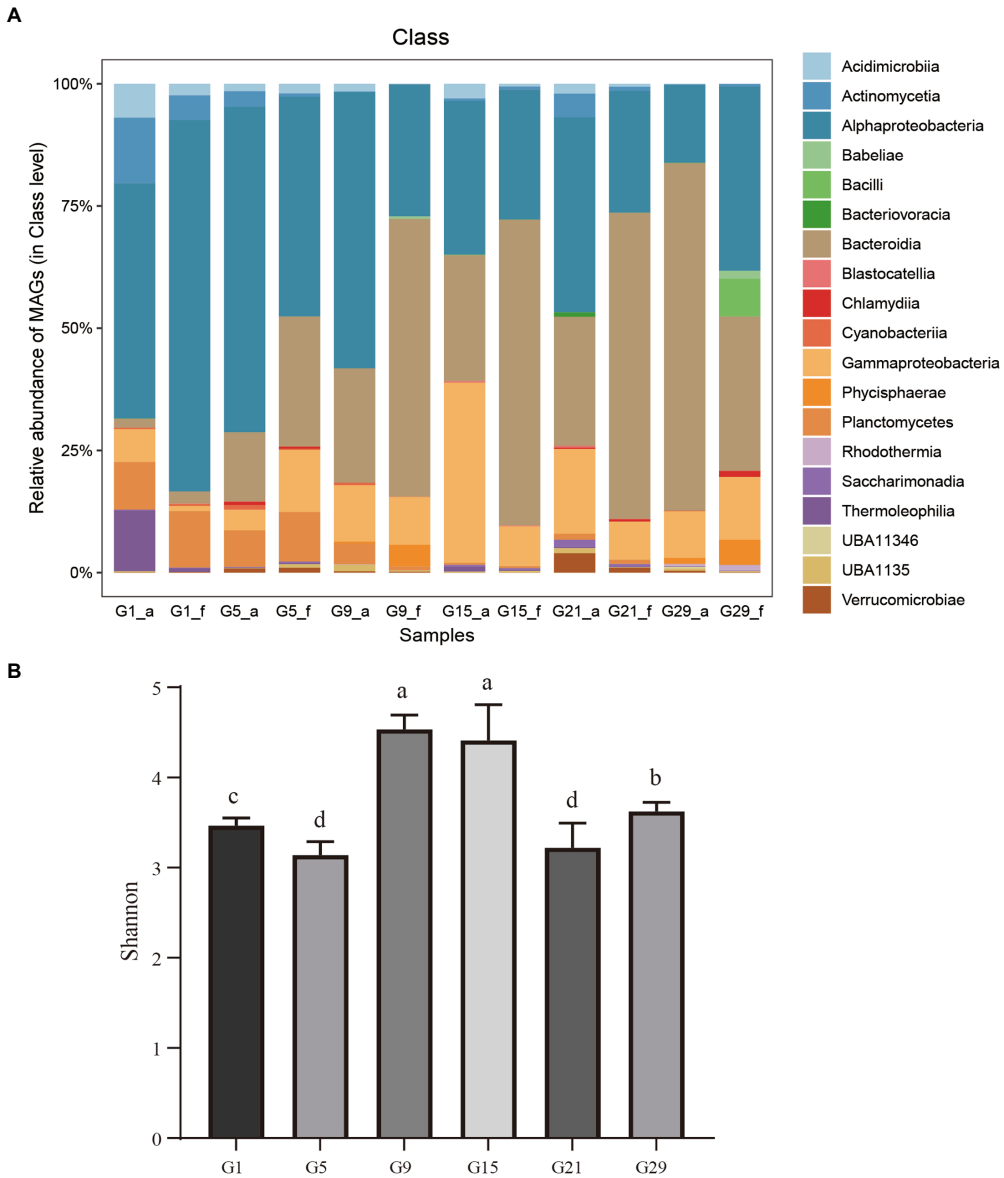
**(A)** The relative abundances of the bacterial MAGs. On the x-axis, the lowercase letter “f” in the sample names indicates the free-living bacteria, and “a” indicates the attached bacteria (*n* = 3). **(B)** Bacterial community alpha diversity at different bloom stages as expressed by the Shannon diversity indices. Values are means of triplicates ± SD. Different lowercase letters (a–d) on the bars represent statistical significance between time points at *p* < 0.05 (Student’s *t*-test).

A heat map was constructed based on the abundance of MAGs to show the relationship and reproducibility among the various samples in the two algal blooms (Supplementary Figure S2). The MAGs exhibited significant differences in their distribution among microbes during the algal bloom process. Furthermore, the species composition among the different bloom stages was significant difference (Supplementary Figure S2; Figure 3A). In the *Skeletonema* bloom, the pre-, onset- and post-stages of the bloom process were dominated by *Thermoleophilia* and *Alphaproteobacteria*, a single *Alphaproteobacteria* taxon, and *Bacteroidia* and *Gammaproteobacteria*, respectively. Unlike the *Skeletonema* bloom, *Gammaproteobacteria* and *Bacteroidia* were dominant during the entire *Phaeocystis* bloom period, although a few fluctuations were observed. An interesting phenomenon is that throughout the complete bloom succession process, *Alphaproteobacteria* showed a gradually decreasing trend, while *Bacteroidia* had a gradually increasing trend.

The Shannon indices showed significant differences between the various bloom stages (Figure 3B). The maximum values Shannon diversity index (4.5 ± 0.3) occurring at the transition period (G9 and G15), both significantly higher than the other stages of the bloom process (*p* < 0.05, Figure 3B).

**Figure 4:**
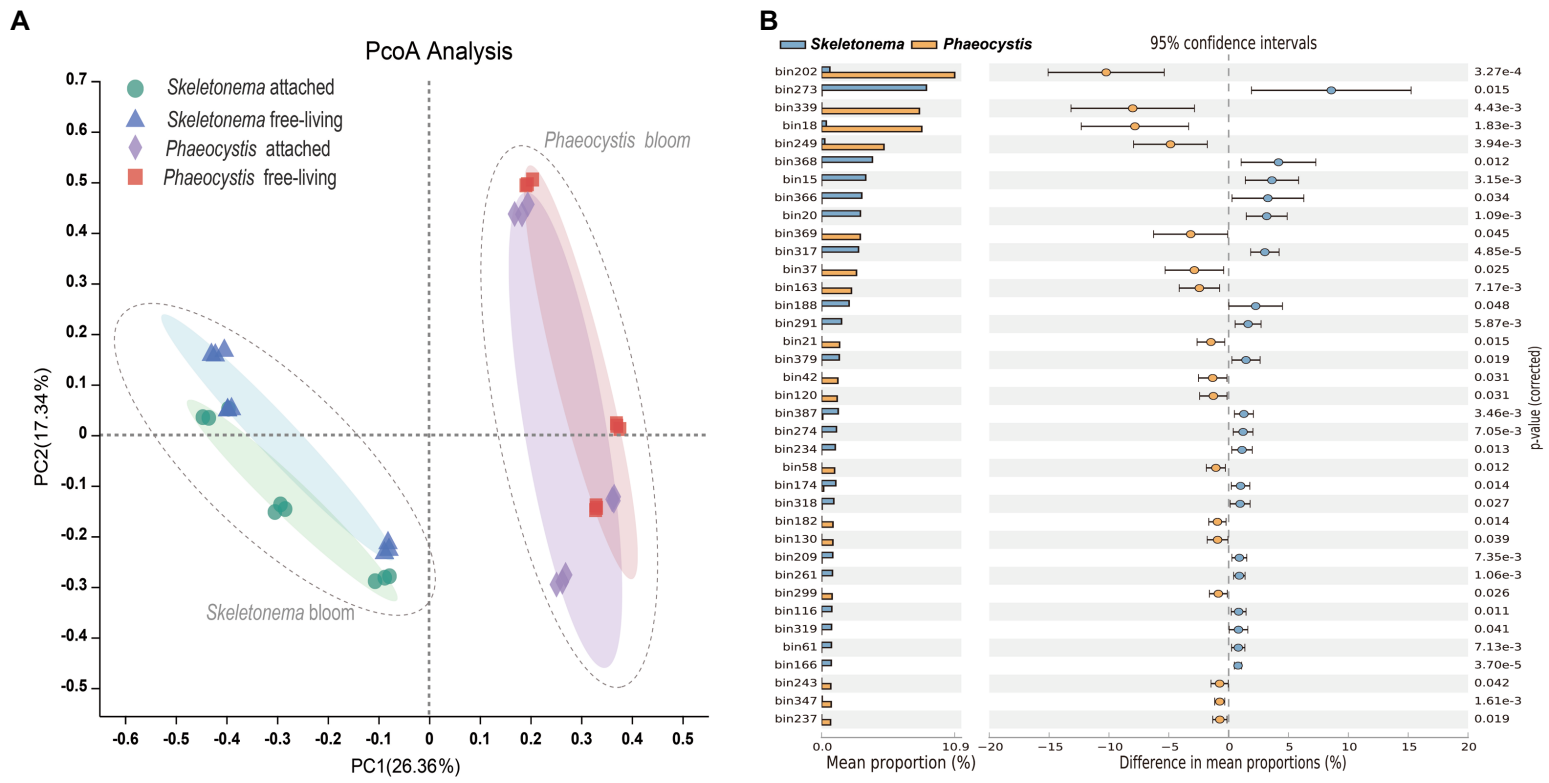
Microbial community composition during bloom succession. **(A)** PCoA plot based on the Bray-Curtis distance for bacterial community composition during the bloom process. **(B)** Differentially abundant MAGs between the *Skeletonema* sp. and *Phaeocystis* sp. blooms using the STAMP software. The *p* value was tested by Student’s *t*-test and corrected using the Bonferroni method.

For the beta diversity, the PCoA plot (based on distributions of bacterial MAGs across points) indicated that samples were divided into *Skeletonema* sp. and *Phaeocystis* sp. groups. All samples were clustered into two groups in the form of *Skeletonema* sp. and *Phaeocystis* sp. bloom stages (Figure 4A). However, no apparent differences were observed between the attached and free-living bacteria. Differentially abundant taxa between the two bloom periods were identified with the STAMP software (Figure 4B). The results showed that the MAGs (mean proportion > 2%) of bin.273, bin.368, bin.15, bin.366, bin.20, bin.317, bin.188, and bin.291 were overrepresented in the *Skeletonema* bloom phase (*p* < 0.05). Among these bins, bin.273, bin.15, bin.20, bin.317, and bin.291 belonged to the family of *Rhodobacteraceae* and constituted 24.58% (relative abundance) of the bacterial community. On the other hand, MAGs (mean proportion > 2%) of bin.202, bin.339, bin.18, bin.249, bin.369, bin.37, and bin.163 were significantly enriched in the *Phaeocystis* sp. phase (*p* < 0.05). Among these, bin.339, bin.249, and bin.369 belonged to the family of *Flavobacteriaceae* and comprised 17.36% (relative abundance) of the bacterial community. The MAG of bin.202 was classified as the genus *Phaeodactylibacter* in the family of *Saprospiraceae* and accounted for 10.47% (relative abundance) of the bacterial community.

### 3.4. Patterns of functional genes in microbial metagenomes

**Figure 5:**
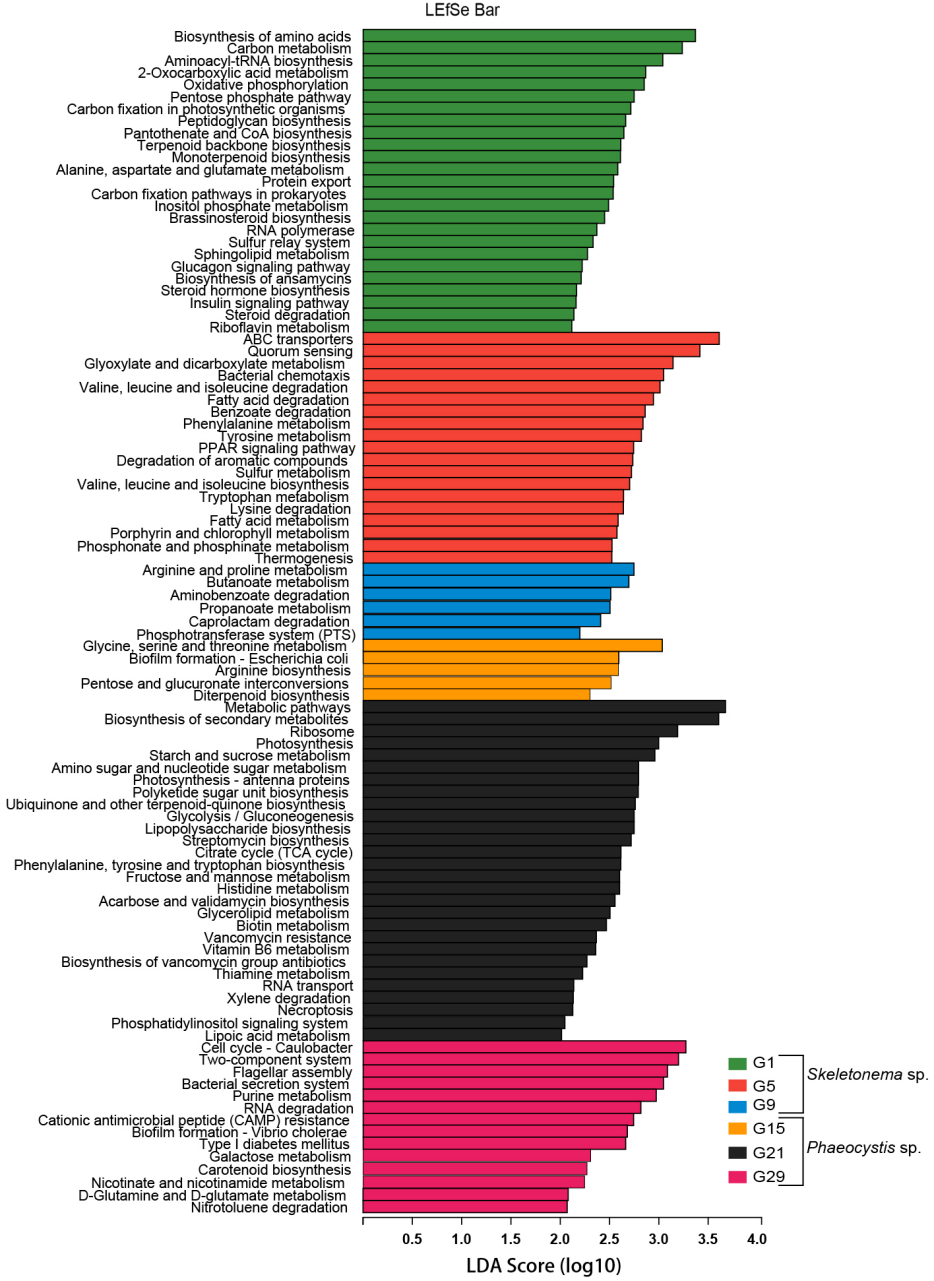
Predicted genes from metagenome related to KEGG pathways differentially represented between the bloom samples identified by LEfSe (LDA > 2, p < 0.05). Different metagenome sampling timepoints were distinguished by different colors. The horizontal bar on the right side indicates that the corresponding KEGG pathway (left side) was significantly enriched at that sampling timepoint.

LEfSe analysis was used to identify differentially abundant metagenome functions (i.e., “functional biomarkers”) among the six stages of the successional process (Figure 5). Taken together, the functions of the algal-related microorganisms had timing concurrent with the bloom phase and seemed to be host-dependent.

**Figure 6:**
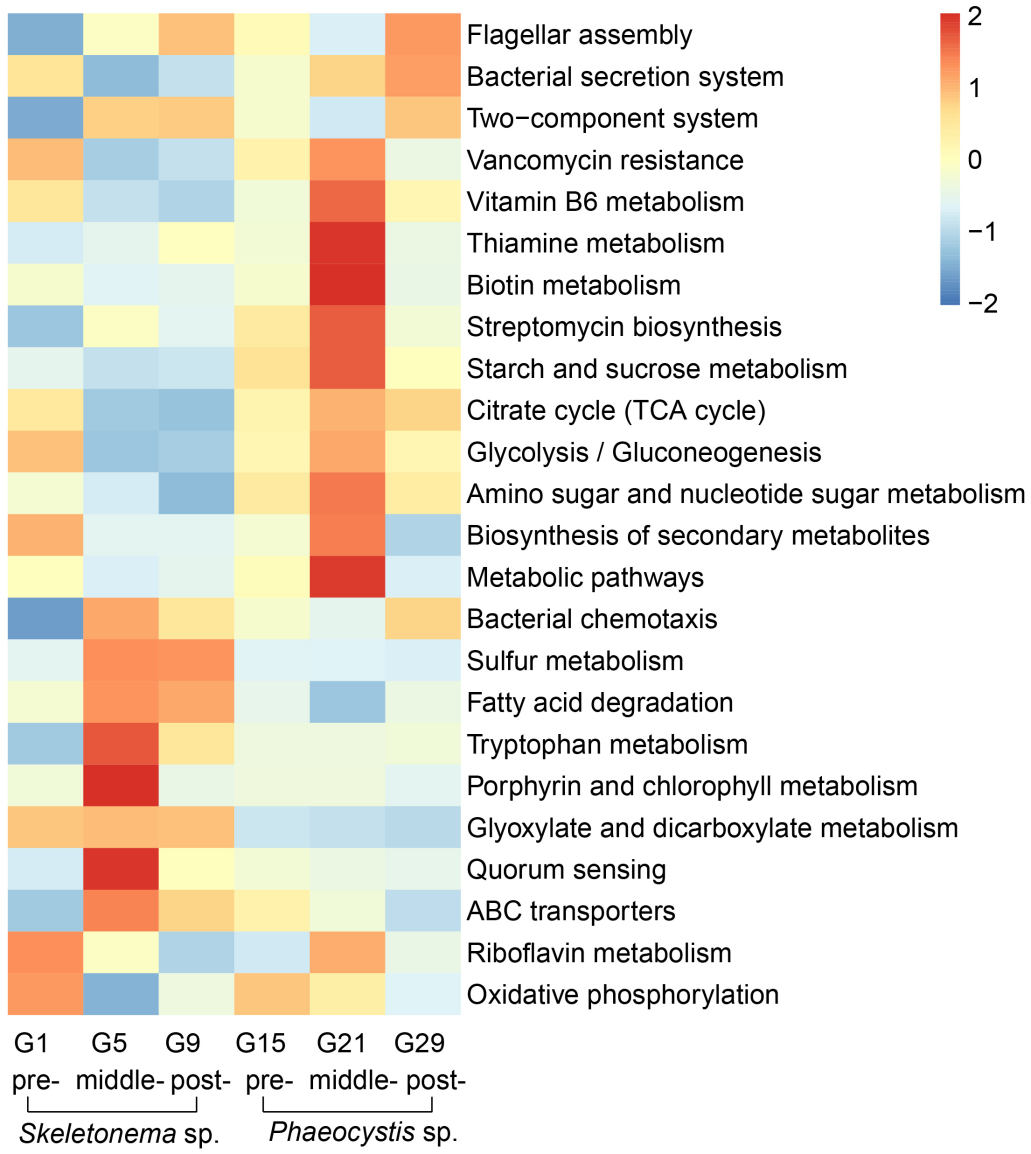
KEGG functional annotation and trends during the algal bloom with z-score values of each KEGG pathway across the rows. The red color indicates high abundance, and blue indicates low abundance.

According to the LEfSe results, some metabolic pathways were significantly enriched during the bloom succession process (Figure 5). The relative abundances of functional genes were presented as a heat map to more clearly understand the trends of these functional marker pathways in each bloom stage (Figure 6). Overall, ABC transporters, quorum sensing (QS), glyoxylate metabolism, bacterial chemotaxis, tryptophan metabolism, fatty acid degradation, sulfur metabolism, porphyrin metabolism, and bacterial chemotaxis were present in the *Skeletonema* bloom stage. On the other hand, metabolic pathways, biosynthesis of secondary metabolites, starch and sucrose metabolism, amino sugar and nucleotide sugar metabolism, glycolysis, citrate cycle (TCA cycle), tryptophan biosynthesis, biotin metabolism, thiamine metabolism, vancomycin resistance, streptomycin biosynthesis, flagellar assembly, and bacterial secretion system were increased in the *Phaeocystis* bloom stage. Essentially, the algal bloom period consisted of time points G5 and G21 that served as stabilization (middle-stages) periods for *Skeletonema* sp. and *Phaeocystis* blooms, respectively. The middle-stage of the *Skeletonema* bloom had the highest abundance of QS, tryptophan metabolism, sulfur metabolism, and porphyrin metabolism. Moreover, tryptophan biosynthesis, biotin metabolism, and thiamine metabolism had the highest abundance in the middle-stage of the *Phaeocystis* bloom. These results confirm that the functional capabilities of microorganisms were strongly related to algal bloom succession.

According to previous studies, signal regulation (QS, IAA) and substance metabolism, especially medium substance between algae and bacteria such as DMSP and VB, are often used as key clues in the study of algae-bacteria interactions (Amin et al., 2015; Zhou et al., 2016; Kessler et al., 2018). In the following sections, we focused on the genes involved in the four substances (DMSP, VB, QS and IAA) commonly studied in algal-bacterial interactions and described their features.

### 3.5. DMSP metabolism

Based on the LEfSe and heatmap results (Figures 5, 6), we further analyzed the relevant pathways related to the conversion of sulfur. As shown in Supplementary Figure S3, some functional genes involved in the sulfur metabolism were species-specific and distributed in different MAG classes. Most MAGs had sulfur-reducing and DMSP-metabolizing abilities. Some MAGs belonging to *Alphaproteobacteria* (60 MAGs) and *Gammaproteobacteria* (13 MAGs) were identified as containing the SOX systems. Regarding the organosulfur DMSP metabolic pathway, most MAGs were identified as containing the cleavage pathway key gene *dddP* (K01271) or *dddL* (K16953), such as MAGs of *Alphaproteobacteria*, and a few had the demethylation pathway key gene *dmdA* (K17486) (Supplementary Figure S3). The MAGs of *Alphaproteobacteria* had a well-established sulfur metabolic pathway, the cleavage and demethylation pathways of DMSP, and a more complete SOX system. In the *Skeletonema* sp. phase, a higher percentage of species participated in DMSP and sulfate reduction, such as bin.58, bin.171, bin.188, and bin.273. In contrast, relatively higher abundances for genes from bin18, bin202, and bin339 were involved in sulfate reduction and the SOX system in the *Phaeocystis* bloom. These results indicate that microorganisms had active sulfur metabolism in the two algal blooms and were performed by different microorganisms.

### 3.6. Synthesis of vitamin B

Functional difference analysis revealed that vitamin synthesis was significantly different between the two bloom stages (Figures 5, 6). To determine the potential role of bacterial communities in providing vitamin B to the host algae, genes involved in the biosynthesis of cobalamin (B_12_), thiamine (B_1_), and biotin (B_7_) in the MAGs were investigated (Supplementary Figure S4). The results showed that the potential for the biosynthesis of vitamins B_1_, B_7_, and B_12_ was widely distributed within the MAGs. Most of the MAGs belonging to *Bacteroidia* (i.e., bin.202, bin.249, bin.339, and bin.369) were capable to synthesize vitamin B_7_; *Gammaproteobacteria* were able to synthesize vitamins B_1_ and B_7_; and *Alphaproteobacteria* (i.e., bin.273, bin.20, and bin.317) encoded the essential genes necessary for the biosynthesis of vitamins B_1_, B_7_, and B_12_. In the *Skeletonema* sp. phase, multiple species were capable in various VB biosynthesis, such as bin.171 for VB_12_, bin.188 for VB_1_ and VB_7_, and bin.20, bin.273, and bin.317 for VB_1_/VB_7_/VB_12_. Bacteria in the *Phaeocystis* bloom were capable of vitamin B_7_ production, including bin.202, bin.249, and bin.339. Our results suggest that the two algal blooms had different VB requirements, and the microorganisms might have provided vitamins for their host.

### 3.7. Signaling process

The indicator functional composition analysis identified QS-related pathways were significantly different during the succession (Figures 5, 6). To obtain the bacterial QS biosynthesis profile during the succession, genomic annotations of the MAGs were performed using sequence homology alignments (Supplementary Figure S5). The abundance of the genes involved in the biosynthesis of different QS molecules varied greatly at the different bloom stages. Overall, most MAGs could synthesize AI-1, DSF, and PQS (Supplementary Figure S5). In addition, few MAGs could synthesize AI-2, such as the MAGs in *Acidimicrobiia* and *Bacteroidia*. Some MAGs belonging to *Alphaproteobacteria*, such as the *Roseobacter* clade strains of bin.273, bin.15, bin.20, bin.317, and bin.291, had more QS signaling synthesis systems and covered almost all of the mentioned QS signaling molecules. In the *Skeletonema* sp. phase, AI-1 synthesis was present in bin.171, bin.188, bin.273, bin.366, and bin.368. Conversely, in the *Phaeocystis* sp. phase, the main taxa (bin.202 and bin.339) were involved in the DSF and PQS signals. Overall, different biosynthetic pathways for the synthesis of QS molecules were present during the different phases of the bloom, with AI-1 pathways mostly represented during the *Skeletonema* sp. phase and AI-2 and other biosynthetic pathways in the *Phaeocystis* sp. phase.

In addition to QS, IAA was also an important signal regulator. Differential functional analysis showed that the genes involved in the tryptophan metabolism and biosynthesis were represented by *Skeletonema* sp. and *Phaeocystis* blooms, respectively (Figures 5, 6). Tryptophan is an important precursor for the synthesis of IAA. We performed a comparative analysis of the IAA biosynthesis pathways in the MAGs (Supplementary Figure S6). The results revealed that most MAGs were capable responsible for IAA biosynthesis, with taxa in *Alphaproteobacteria* having a relatively complete IAA synthesis pathway. Some MAGs belonging to *Acidimicrobiia* (i.e., bin.73, bin.293, bin.191, and bin.306) and *Alphaproteobacteria* (i.e., bin.20 and bin.278) had the complete pathways to convert tryptophan to indole-3-acetamide (required gene K00466) and then to IAA (required gene K01426). The bin.273 and some MAGs in the class of *Bacteroidia* were regarded as containing the genes involved in the biosynthesis of IAA, where tryptamine (required gene K01593) was converted to indole-3-acetaldehyde (required gene K00274) and then to IAA (required gene K00128). In the two algal blooms, the genes involved in the biosynthesis of the IAA appeared in the pre-and onset stage and were identified as belonging to different bacteria (bin.188, bin.171, bin.273, and bin.368 in the *Skeletonema* sp. phase, and bin.202 and bin.339 in the *Phaeocystis* sp. phase). Based on the fact that IAA appeared in the early stage of algal bloom shows that IAA could play a certain role in promoting the formation of algal blooms.

## 4. Discussion

*Skeletonema* sp. and *Phaeocystis* sp. play important roles in elemental cycles, marine food webs, and potential climate change (Hamm, 2000; Verity et al., 2007; Kooistra et al., 2008). On the other hand, they often cause algal blooms, and the formation of *Phaeocystis* blooms has been frequently observed to accompany or succeed the diatom blooms (Larsen et al., 2004; Grattepanche et al., 2011). Similar to previous reports, the succession of algal species from *Skeletonema* sp. to *Phaeocystis* sp. was observed during this artificial algal bloom study (Grattepanche et al., 2010, 2011; Liu et al., 2022). The highest densities of the two blooms induced in this study were close to or even exceeded the previously reported peak algal densities during natural blooms (Duyl et al., 1998; Zhou et al., 2020a,b; Xu et al., 2022), indicating that we successfully simulated natural blooms. However, the regulation mechanism of the bloom succession at microbial level (bacterial community and metabolic potential) is still unknown. This study explored the microbial composition and metabolic potential at different bloom stages from *Skeletonema* sp. to *Phaeocystis* sp. using metagenomic sequencing data. We observed distinct temporal patterns for the microbial communities and function, which extended our understanding of the bloom succession mechanism from a microbial perspective.

**Figure 7:**
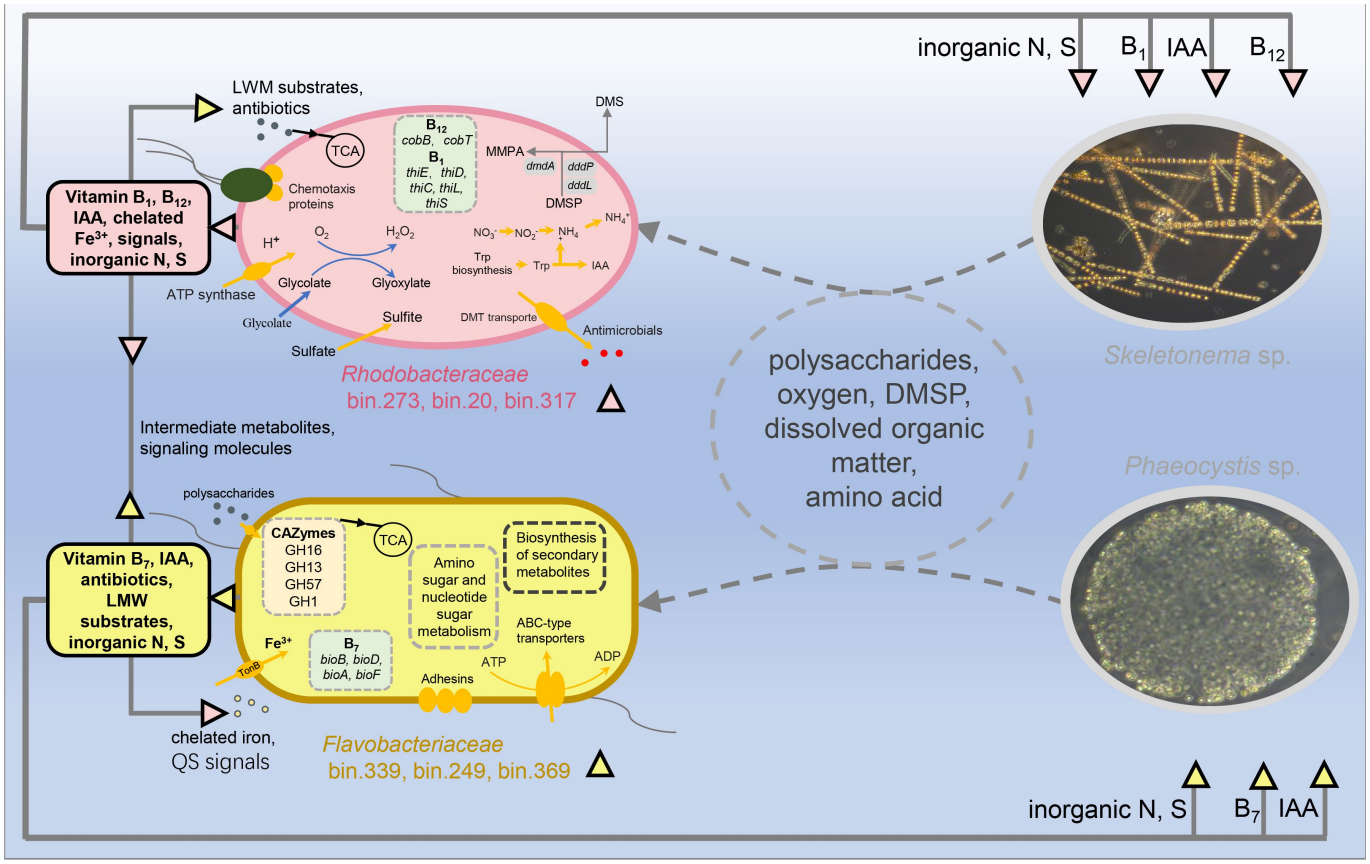
Potential metabolic interactions between the algae blooms and the reconstructed genomes of the two bacterial lineages, *Flavobacteriaceae* and *Rhodobacteraceae*. Colored triangles indicate metabolites sourced from *Flavobacteriaceae* (orange) and *Rhodobacteraceae* (pink), while arrow directions indicate predicted exchanges.

It was previously mentioned that host algae and their associated microbes interact and influence each other, jointly shaping the symbiotic composition (Ramanan et al., 2016). Many studies confirmed that algae-associated bacteria tend to be dominated by specific members of *Bacteroidetes*, *Alphaproteobacteria*, and *Gammaproteobacteria* (Buchan et al., 2014). This was hypothesized to be because these bacterial groups’ metabolic properties allow them to readily respond to algal-secreted nutrient fluctuations during the bloom period (Buchan et al., 2014). In addition, the composition and metabolic function of microorganisms, in turn, regulate the growth of host algae (Kim et al., 2014; Amin et al., 2015). This may be an internal driving force for the host succession. Moreover, the bacterial MAGs across samples suggested that algae-associated bacteria exhibited partly host dependencies to some extent like previously reported by Pinto et al. (2021). Therefore, we combined the free-living and attached bacteria to determine a change in composition. We identified the microbial markers in the communities of the two blooms (Figures 4B, 7). The results showed that bin.273, bin.15, bin.20, bin.317, and bin.291 dominated in the *Skeletonema* bloom phase, belonging to *Rhodobacteraceae*. In contrast, bin.339, bin.249, and bin.369 belonged to *Flavobacteriaceae* were significantly enriched in the *Phaeocystis* sp. phase. These results suggest that microbial communities and function respond distinctly to the succession of host algae.

Several studies examining the bacterial communities of diatom cultures or diatom blooms have demonstrated that *Rhodobacteraceae* are the main associated heterotrophic bacteria (Grossart et al., 2005; Amin et al., 2012), consistent with our results. Zecher et al. (2020) suggested that *Rhodobacteraceae* can degrade dissolved organic nitrogen (i.e., monomethylamine) to produce ammonium, which then the ammonium serves as a nitrogen source for the diatoms. This process might help regulate organism-specific metabolic interactions to establish and stabilize associations with photoautotrophic diatoms (Rambo et al., 2020). The bacteria belonging to *Rhodobacteraceae* are prominent and have been recorded as abundant members of planktonic communities during diatom blooms (Morris et al., 2006). Like *Rhodobacteraceae*, *Flavobacteriaceae* are also dominant members in phytoplankton bloom events (Buchan et al., 2014). During the bloom period, the abundance of *Flavobacteriaceae* was usually highest in the decay/death phase. It is important to note that there are many possible hypotheses for the appearance of *Flavobacteriaceae*. For example, *Flavobacteriaceae* might be regarded as an opportunist by probably benefiting from the exudates of dead *Skeletonema* or the extracellular organic matter secreted by *Phaeocystis*. In some previous studies, it has been proposed that the main role of *Flavobacteriaceae* is to convert high molecular compounds to low molecular compounds (Riemann et al., 2000; Teeling et al., 2012). Even so, *Flavobacteriaceae* and *Phaeocystis* may have a mutually beneficial symbiotic relationship because *Flavobacteriaceae* maintained a higher abundance in the pre-stage of *Phaeocystis* bloom (Figures 4B, 7). Recent evidence documented that Flavobacterial exudates can change the morphology of algal cells, which causes algal cells to become larger and secrete more carbohydrates (Bartolek et al., 2022). Current phylogenetic analyses of the marine microbiome by experimental and natural phytoplankton blooms suggest that members of *Flavobacteriaceae* (i.e., *Polaribacter*, *Robiginitalea*) are commonly associated with the bloom development of the *Phaeocystis* population (Wemheuer et al., 2015; Li et al., 2020). In this work, we also found that the MAGs of bin.339, bin.249, and bin.369, associated with the *Phaeocystis* bloom, belonged to *Flavobacteriaceae*. These suggest that *Flavobacteriaceae* may have functional differences for different algae.

An interesting phenomenon observed during the succession transitional period (G13–G15) was the dominance of the *Roseobacter* in bacterial communities. The *Roseobacter* clade have the genetic potential for bacteriochlorophyll-related aerobic anoxygenic phototrophic (AAnP) metabolism, degrading algae-derived compounds (e.g., DMSP) along with the production of the climate-relevant gas, dimethyl sulfide (DMS) (Cunliffe, 2011). DMS can act as a chemical signal that affects the bacterial and phytoplankton community composition (Shemi et al., 2021; Teng et al., 2021). *Roseobacter* also produces iron chelators, which act as public substances that help algae or bacteria cope with iron limitations and are important mediators in algae-bacteria interactions (Amin et al., 2009; Kazamia et al., 2018).

Accordant with the results mentioned above, we suggest that microbes are ecological regulators for algae that affect the state of the host algae and promote their succession by nutrient exchange and signal communication. However, it is worth noting that many challenges still exist in directly linking bacterial lineages to algal succession (Zhou et al., 2020a). The isolation and purification of bacteria are needed to achieve binary co-cultures of algae and bacteria to elucidate the precise contribution of microorganisms to bloom successions.

To gain more insight into the impact of these bacteria on algal bloom succession, the MAGs were reconstructed and functionally annotated (Figure 5). We observed noticeable changes in microbial metabolic capacities in the two algal bloom successions, such as signal molecules (QS, IAA) and nutrients substance (DMSP, VB). These compounds are often used as key clues in the study of algae-bacteria interactions (Amin et al., 2015; Zhou et al., 2016; Kessler et al., 2018).

Complex inter- or intra- interactions are key factors affecting the dynamics of blooms (Amin et al., 2012). The interactions between algae and bacteria could be developed through informative chemicals by controlling physiological activities and gene expression (Amin et al., 2015). Quorum sensing is a specific language of chemical signals and plays an ecological role in algae-bacteria interactions (Zhou et al., 2016). Our previous studies have shown that the QS systems regulated bacterial community composition and function during the bloom event (Huang et al., 2018; Zhu et al., 2022). This work showed that several MAGs belonging to *Alphaproteobacteria* had more QS signaling synthesis systems and covered almost all the mentioned QS signaling molecular synthesis systems (Supplementary Figure S5). The main class of quorum-sensing signals produced by Gram-negative bacteria is one of the AI-1, acyl-homoserine lactones (AHLs). Several studies have shown that AHLs directly affect diatom growth and metabolism [reviewed by Qiao et al. (2022)]. In addition, QS signals were involved in nutrient acquisition, recycling, and bacterial community structure (reviewed by Dow, 2021). In this work, we also observed close relevance between the QS genes and algal succession. The high-abundance bacteria (bin.15, bin.20, bin.273, and bin.317) of the *Skeletonema* bloom period were mainly capable of the AHL, DSF, and PQS signal communication, while the high-abundance bacteria (bin.339, bin.249, and bin.369) of the *Phaeocystis* bloom period were primarily DSF and PQS. Taken together, these results reconfirm that QS may participate in affecting bloom succession by regulating the microbial behavior and available algal nutrients (Figure 7).

In addition, members of *Alphaproteobacteria*, such as the *Roseobacter* clades, can synthesize IAA to promote diatom cell division (Moran et al., 2007; Amin et al., 2015). IAA was previously identified as a key component of the algae-bacteria chemical crosstalk, and bacterially-produced IAA increases algal yields (Segev et al., 2016). Here, the symbiotic bacteria responsible for IAA synthesis were present in both bloom periods, but they differed greatly at the taxonomic level (Supplementary Figure S6). In the *Skeletonema* bloom, MAGs in *Alphaproteobacteria* were responsible for IAA synthesis, while MAGs in the *Bacteroidia* were identified in the *Phaeocystis* bloom. Thus, the formation and succession of blooms from *Skeletonema* sp. to *Phaeocystis* sp. might be controlled by bacterial communication and related behavior.

Along with signal regulation, microorganisms drive metabolite transformations. For example, some phytoplankton produces large amounts of the organic sulfur compound DMSP during bloom events, which can be converted to the gas DMS *via* the bacterial cleavage pathway (Moran et al., 2012). Both diatoms and *Phaeocystis* are important in the production of DMS (Jiang et al., 2014). Evidence suggests that bacterial demethylation predominates over cleavage in the ocean, as approximately 90% of DMSP is processed through this pathway (Kiene et al., 1999, 2000). In the present work, we found the key genes *dddP* (K01271) or *dddL* (K16953) from *Alphaproteobacteria* involved in this pathway in bin.15, bin.20, bin.273, and bin.317 in the *Skeletonema* bloom period. In addition, the demethylation pathway key gene *dmdA* (K17486) was also identified in bin.317, bin.291, and bin.20 (Supplementary Figure S3). DMSP has dual roles: it serves as a source of carbon and sulfur for bacteria and a chemical signal that attracts motile bacteria to their host algae (Seymour et al., 2010; Curson et al., 2011). Bacteria capable of metabolizing DMSP as a source of carbon and/or sulfur are exceptionally responsive to these informative chemotactic chemicals (Seymour et al., 2010).

Like DMSP, vitamin B is an important biotin for host algae. Most eukaryotic phytoplankton are auxotrophs of VB because they require exogenous B-family vitamins to maintain their growth (Peperzak et al., 2000; Droop, 2007). Many studies have identified algal species that require different combinations of the three B vitamins: B_12_, B_1_, and B_7_ (Croft et al., 2005). During this experiment, the genes involved in the biosynthesis of vitamin B_12_ were represented in the *Skeletonema* bloom stage, while the genes involved in the biosynthesis of biotin and thiamine were remarkably present in the *Phaeocystis* bloom stage (Supplementary Figure S4). This suggests symbiotic bacteria from different algae has different synthetic abilities for vitamin B. Bacteroidia, Gammaproteobacteria, and Alphaproteobacteria were potentially responsible for the metabolism of other B vitamins (Supplementary Figure S4). Our results imply that algae may be provided with different B vitamins from the symbiotic bacteria.

## 5. Conclusion

This study provides a *de novo* metagenomic reconstruction of individual genomes from bacterial communities and offers novel insight into how microbial structure–function covariation affects algal bloom succession. The conceptual diagram of the overall process is shown in Figure 7. The diagram indicates that bacterial communities exhibited clear changes in diversity and composition during algal bloom successions. The potential keystone taxa (e.g., “biomarkers”) associated with different bloom hosts were identified, with *Rhodobacteraceae* and *Flavobacteriaceae* being associated with of *Skeletonema* and *Phaeocystis*, respectively. These taxa were further identified as possibly involved in vitamin synthesis, IAA production, quorum-sensing signals, and DMSP metabolism. Additionally, some bacteria had a broad metabolic spectrum. The results confirm our hypothesis that the changes in bacterial community structure and function are associated with the succession of algae from *Skeletonema* sp. to *Phaeocystis* sp., which helps us better understand the algal bloom succession from a micro-ecological viewpoint. However, it should be pointed out that the present findings are limited to inferring from the correlation between the host algae and associated bacteria, and no definite conclusion can be drawn on the causal relationship between them. Future research will seek to unravel the mechanisms affecting interspecies and cross-kingdom interactions of the bacterial communities associated with these bloom successions by isolating and culturing algae associated bacterial strains.

## Data availability statement

The datasets presented in this study can be found in online repositories. The names of the repository/repositories and accession number(s) can be found at: https://www.ncbi.nlm.nih.gov/genbank/, PRJNA773615.

## Ethics statement

Ethical review and approval were not required for the study on human participants following the local legislation and institutional requirements. Written informed consent for participation was not required for this study following the national legislation and the institutional requirements.

## Author contributions

JMZ and JNZ: conceptualization, investigation, writing-original draft preparation, statistical analysis, software application, and figures drawing. ST and KC: data analysis. GC and ZC: read and revised the manuscript, funding acquisition, and project administration. All authors contributed to the article and approved the submitted version.

## Funding

This work was supported by the National Natural Science Foundation of China (41976126), Guangdong Basic and Applied Basic Research Foundation (2020B1515120012), Shenzhen Science and Technology Program (Grant No. RCJC20200714114433069), S&T Projects of Shenzhen Science and Technology Innovation Committee (KCXFZ202110201633557022, JCYJ20200109142818589, JCYJ20200109142822787, and WDZC20200819173345002), Project of Shenzhen Municipal Bureau of Planning and Natural Resources (Grant No. [2021]735–927), Shenzhen-Hong Kong-Macao Joint S&T Project (pending number 202205303000176), as well as Shandong Provincial Natural Science Foundation, China (ZR2020MD081).

## Conflict of interest

The authors declare that the research was conducted in the absence of any commercial or financial relationships that could be construed as a potential conflict of interest.

## Publisher’s note

All claims expressed in this article are solely those of the authors and do not necessarily represent those of their affiliated organizations, or those of the publisher, the editors and the reviewers. Any product that may be evaluated in this article, or claim that may be made by its manufacturer, is not guaranteed or endorsed by the publisher.

## Footnotes

^1^ www.illumina.com

^2^ https://github.com/wwood/CoverM

^3^ https://github.com/geronimp/enrichM

^4^ https://itol.embl.de

^5^ https://huttenhower.sph.harvard.edu/galaxy/

## References

[ref1] Amin S. A. Green D. H. Hart M. C. Küpper F. C. Sunda W. G. Carrano C. J. (2009). Photolysis of iron–siderophore chelates promotes bacterial–algal mutualism. Proc. Natl. Acad. Sci. U. S. A. 106, 17071–17076. doi: 10.1073/pnas.0905512106, PMID: 19805106 PMC2761308

[ref2] Amin S. A. Hmelo L. R. van Tol H. M. Durham B. P. Carlson L. T. Heal K. R.. (2015). Interaction and signalling between a cosmopolitan phytoplankton and associated bacteria. Nature 522, 98–101. doi: 10.1038/nature14488, PMID: 26017307

[ref3] Amin S. A. Parker M. S. Armbrust E. V. (2012). Interactions between diatoms and bacteria. Microbiol. Mol. Biol. Rev. 76, 667–684. doi: 10.1128/MMBR.00007-12, PMID: 22933565 PMC3429620

[ref4] Bartolek Z. Creveld S. G. Coesel S. Cain K. R. Schatz M. Morales R.. (2022). Flavobacterial exudates disrupt cell cycle progression and metabolism of the diatom *Thalassiosira pseudonana*. ISME J. 16, 2741–2751. doi: 10.1038/s41396-022-01313-9, PMID: 36104452 PMC9666458

[ref5] Buchan A. LeCleir G. R. Gulvik C. A. González J. M. (2014). Master recyclers: features and functions of bacteria associated with phytoplankton blooms. Nat. Rev. Microbiol. 12, 686–698. doi: 10.1038/nrmicro3326, PMID: 25134618

[ref6] Buchfink B. Xie C. Huson D. H. (2015). Fast and sensitive protein alignment using DIAMOND. Nat. Methods 12, 59–60. doi: 10.1038/nmeth.3176, PMID: 25402007

[ref7] Chaumeil P. A. Mussig A. J. Hugenholtz P. Parks D. H. (2019). GTDB-Tk: a toolkit to classify genomes with the genome taxonomy database. Bioinformatics 36, 1925–1927. doi: 10.1093/bioinformatics/btz848, PMID: 31730192 PMC7703759

[ref8] Chen S. Zhou Y. Chen Y. Gu J. (2018). Fastp: an ultra-fast all-in-one FASTQ preprocessor. Bioinformatics 34, i884–i890. doi: 10.1093/bioinformatics/bty560, PMID: 30423086 PMC6129281

[ref9] Croft M. T. Lawrence A. D. Raux Deery E. Warren M. J. Smith A. G. (2005). Algae acquire vitamin B_12_ through a symbiotic relationship with bacteria. Nature 438, 90–93. doi: 10.1038/nature04056, PMID: 16267554

[ref10] Cui Y. Chun S. J. Baek S. S. Baek S. H. Kim P. J. Son M.. (2020). Unique microbial module regulates the harmful algal bloom (*Cochlodinium polykrikoides*) and shifts the microbial community along the southern coast of Korea. Sci. Total Environ. 721:137725. doi: 10.1016/j.scitotenv.2020.137725, PMID: 32182460

[ref11] Cunliffe M. (2011). Correlating carbon monoxide oxidation with cox genes in the abundant marine *Roseobacter* clade. ISME J. 5, 685–691. doi: 10.1038/ismej.2010.170, PMID: 21068776 PMC3105738

[ref12] Curson A. R. J. Todd J. D. Sullivan M. J. Johnston A. W. B. (2011). Catabolism of dimethylsulphoniopropionate: microorganisms, enzymes and genes. Nat. Rev. Microbiol. 9, 849–859. doi: 10.1038/nrmicro2653, PMID: 21986900

[ref13] Dixon P. (2003). VEGAN, a package of R functions for community ecology. J. Veg. Sci. 14, 927–930. doi: 10.1111/j.1654-1103.2003.tb02228.x

[ref14] Dow L. (2021). How do quorum-sensing signals mediate algae-bacteria interactions? Microorganisms 9:1391. doi: 10.3390/microorganisms9071391, PMID: 34199114 PMC8307130

[ref15] Droop M. R. (2007). Vitamins, phytoplankton and bacteria: symbiosis or scavenging? J. Plankton Res. 29, 107–113. doi: 10.1093/plankt/fbm009

[ref16] Duyl F. C. Gieskes W. W. C. Kop A. J. Lewis W. E. (1998). Biological control of short-term variations in the concentration of DMSP and DMS during a *Phaeocystis* spring bloom. J. Sea Res. 40, 221–231. doi: 10.1016/S1385-1101(98)00024-0

[ref17] Dwivedi R. Rafeeq M. Smitha B. R. Padmakumar K. B. Thomas L. C. Sanjeevan V. N.. (2015). Species identification of mixed algal bloom in the northern Arabian Sea using remote sensing techniques. Environ. Monit. Assess. 187:51. doi: 10.1007/s10661-015-4291-2, PMID: 25638059

[ref18] Fu L. Niu B. Zhu Z. Wu S. Li W. (2012). CD-HIT: accelerated for clustering the next-generation sequencing data. Bioinformatics 28, 3150–3152. doi: 10.1093/bioinformatics/bts565, PMID: 23060610 PMC3516142

[ref19] Fuentes J. L. Garbayo I. Cuaresma M. Montero Z. González-del-Valle M. Vílchez C. (2016). Impact of microalgae-bacteria interactions on the production of algal biomass and associated compounds. Mar. Drugs 14:100. doi: 10.3390/md14050100, PMID: 27213407 PMC4882574

[ref20] Grattan L. M. Holobaugh S. Morris J. G. (2016). Harmful algal blooms and public health. Harmful Algae 57, 2–8. doi: 10.1016/j.hal.2016.05.003, PMID: 27616971 PMC5016795

[ref21] Grattepanche J. D. Breton E. Brylinski J. M. Lecuyer E. Christaki U. (2010). Succession of primary producers and micrograzers in a coastal ecosystem dominated by *Phaeocystis globosa* blooms. J. Plankton Res. 33, 37–50. doi: 10.1093/plankt/fbq097

[ref22] Grattepanche J. D. Vincent D. Breton E. Christaki U. (2011). Microzooplankton herbivory during the diatom–*Phaeocystis* spring succession in the eastern English Channel. J. Exp. Mar. Biol. Ecol. 404, 87–97. doi: 10.1016/j.jembe.2011.04.004

[ref23] Grossart H. P. Levold F. Allgaier M. Simon M. Brinkhoff T. (2005). Marine diatom species harbour distinct bacterial communities. Environ. Microbiol. 7, 860–873. doi: 10.1111/j.1462-2920.2005.00759.x, PMID: 15892705

[ref24] Hamm C. E. (2000). Architecture, ecology and biogeochemistry of *Phaeocystis* colonies. J. Sea Res. 43, 307–315. doi: 10.1016/S1385-1101(00)00014-9

[ref25] Huang X. Zhu J. Cai Z. Lao Y. Jin H. Yu K.. (2018). Profiles of quorum sensing (QS)-related sequences in phycospheric microorganisms during a marine dinoflagellate bloom, as determined by a metagenomic approach. Microbiol. Res. 217, 1–13. doi: 10.1016/j.micres.2018.08.015, PMID: 30384903

[ref26] Jiang M. Borkman D. G. Scott Libby P. Townsend D. W. Zhou M. (2014). Nutrient input and the competition between *Phaeocystis pouchetii* and diatoms in Massachusetts Bay spring bloom. J. Mar. Syst. 134, 29–44. doi: 10.1016/j.jmarsys.2014.02.011

[ref27] Kanehisa M. Sato Y. Kawashima M. Furumichi M. Tanabe M. (2015). KEGG as a reference resource for gene and protein annotation. Nucleic Acids Res. 44, D457–D462. doi: 10.1093/nar/gkv1070, PMID: 26476454 PMC4702792

[ref28] Kazamia E. Sutak R. Paz Yepes J. Dorrell R. G. Vieira F. R. J. Mach J.. (2018). Endocytosis-mediated siderophore uptake as a strategy for Fe acquisition in diatoms. Sci. Adv. 4:eaar4536. doi: 10.1126/sciadv.aar4536, PMID: 29774236 PMC5955625

[ref29] Keller A. A. Riebesell U. (1989). Phytoplankton carbon dynamics during a winter-spring diatom bloom in an enclosed marine ecosystem: primary production, biomass and loss rates. Mar. Biol. 103, 131–142. doi: 10.1007/BF00391071

[ref30] Kessler R. W. Weiss A. Kuegler S. Hermes C. Wichard T. (2018). Macroalgal–bacterial interactions: Role of dimethylsulfoniopropionate in microbial gardening by *Ulva* (Chlorophyta). Mol. Ecol. 27, 1808–1819. doi: 10.1111/mec.14472 29290092

[ref31] Kiene R. P. Linn L. J. Bruton J. A. (2000). New and important roles for DMSP in marine microbial communities. J. Sea Res. 43, 209–224. doi: 10.1016/S1385-1101(00)00023-X

[ref32] Kiene R. P. Linn L. J. González J. Moran M. A. Bruton J. A. (1999). Dimethylsulfoniopropionate and methanethiol are important precursors of methionine and protein-sulfur in marine bacterioplankton. Appl. Environ. Microbiol. 65, 4549–4558. doi: 10.1128/AEM.65.10.4549-4558.1999, PMID: 10508088 PMC91606

[ref33] Kim B. H. Ramanan R. Cho D. H. Oh H. M. Kim H. S. (2014). Role of *rhizobium*, a plant growth promoting bacterium, in enhancing algal biomass through mutualistic interaction. Biomass Bioenergy 69, 95–105. doi: 10.1016/j.biombioe.2014.07.015

[ref34] Kooistra W. H. Sarno D. Balzano S. Gu H. Andersen R. A. Zingone A. J. P. (2008). Global diversity and biogeography of *Skeletonema* species (Bacillariophyta). Protist 159, 177–193. doi: 10.1016/j.protis.2007.09.004, PMID: 18042429

[ref35] Larsen A. Flaten G. A. F. Sandaa R. A. Castberg T. Thyrhaug R. Erga S. R.. (2004). Spring phytoplankton bloom dynamics in Norwegian coastal waters: microbial community succession and diversity. Limnol. Oceanogr. 49, 180–190. doi: 10.4319/lo.2004.49.1.0180

[ref36] Li R. Li Y. Kristiansen K. Wang J. (2008). SOAP: short oligonucleotide alignment program. Bioinformatics 24, 713–714. doi: 10.1093/bioinformatics/btn025, PMID: 18227114

[ref37] Li D. Liu C. M. Luo R. Sadakane K. Lam T. W. (2015). MEGAHIT: an ultra-fast single-node solution for large and complex metagenomics assembly via succinct de Bruijn graph. Bioinformatics 31, 1674–1676. doi: 10.1093/bioinformatics/btv033, PMID: 25609793

[ref38] Li N. Zhao H. Jiang G. Xu Q. Tang J. Li X.. (2020). Phylogenetic responses of marine free-living bacterial community to *Phaeocystis globosa* bloom in Beibu gulf, China. Front. Microbiol. 11:1624. doi: 10.3389/fmicb.2020.01624, PMID: 32765460 PMC7378386

[ref39] Liu Y. Li L. Zhai X. Zhou J. Ye P. Huang S. (2022). Analysis of the bloom caused by colonial *Phaeocystis globosa* in Mirs Bay. J. Trop. Oceanogr, 41: 164–171

[ref40] Livanou E. Lagaria A. Psarra S. Lika K. (2017). Dissolved organic matter release by phytoplankton in the context of the dynamic energy budget theory. Biogeosci. Discuss. 2017, 1–33. doi: 10.5194/bg-2017-426

[ref41] Morabito S. Silvestro S. Faggio C. (2018). How the marine biotoxins affect human health. Nat. Prod. Res. 32, 621–631. doi: 10.1080/14786419.2017.1329734 28532167

[ref42] Moran M. A. Belas R. Schell M. A. González J. M. Sun F. Sun S.. (2007). Ecological genomics of marine roseobacters. Appl. Environ. Microbiol. 73, 4559–4569. doi: 10.1128/AEM.02580-06, PMID: 17526795 PMC1932822

[ref43] Moran M. A. Reisch C. R. Kiene R. P. Whitman W. B. (2012). Genomic insights into bacterial DMSP transformations. Annu. Rev. Mar. Sci. 4, 523–542. doi: 10.1146/annurev-marine-120710-100827, PMID: 22457986

[ref44] Morris R. M. Longnecker K. Giovannoni S. J. (2006). *Pirellula* and OM43 are among the dominant lineages identified in an Oregon coast diatom bloom. Environ. Microbiol. 8, 1361–1370. doi: 10.1111/j.1462-2920.2006.01029.x, PMID: 16872400

[ref45] Needham D. M. Fuhrman J. A. (2016). Pronounced daily succession of phytoplankton, archaea and bacteria following a spring bloom. Nat. Microbiol. 1:16005. doi: 10.1038/nmicrobiol.2016.5, PMID: 27572439

[ref46] Nguyen L. T. Schmidt H. A. Haeseler A. Quang B. (2014). IQ-TREE: a fast and effective stochastic algorithm for estimating maximum-likelihood phylogenies. Mol. Biol. Evol. 32, 268–274. doi: 10.1093/molbev/msu300, PMID: 25371430 PMC4271533

[ref47] Noguchi H. Park J. Takagi T. (2006). MetaGene: prokaryotic gene finding from environmental genome shotgun sequences. Nucleic Acids Res. 34, 5623–5630. doi: 10.1093/nar/gkl723, PMID: 17028096 PMC1636498

[ref48] Olm M. R. Brown C. T. Brooks B. Banfield J. F. (2017). dRep: a tool for fast and accurate genomic comparisons that enables improved genome recovery from metagenomes through de-replication. ISME J. 11, 2864–2868. doi: 10.1038/ismej.2017.126, PMID: 28742071 PMC5702732

[ref49] Parks D. H. Tyson G. W. Hugenholtz P. Beiko R. G. (2014). STAMP: statistical analysis of taxonomic and functional profiles. Bioinformatics 30, 3123–3124. doi: 10.1093/bioinformatics/btu494, PMID: 25061070 PMC4609014

[ref50] Patro R. Duggal G. Love M. I. Irizarry R. A. Kingsford C. (2017). Salmon provides fast and bias-aware quantification of transcript expression. Nat. Methods 14, 417–419. doi: 10.1038/nmeth.4197, PMID: 28263959 PMC5600148

[ref51] Peperzak L. Gieskes W. W. C. Duin R. Colijn F. (2000). The vitamin B requirement of *Phaeocystis globosa* (Prymnesiophyceae). J. Plankton Res. 22, 1529–1537. doi: 10.1093/plankt/22.8.1529

[ref52] Pinto J. Lami R. Krasovec M. Grimaud R. Urios L. Lupette J.. (2021). Features of the opportunistic behaviour of the marine bacterium *Marinobacter algicola* in the microalga *Ostreococcus tauri* phycosphere. Microorganisms 9:1777. doi: 10.3390/microorganisms9081777, PMID: 34442856 PMC8399681

[ref53] Qiao Z. Li J. Qin S. (2022). Bioactive compounds for quorum sensing signal-response systems in marine phycosphere. J. Mar. Sci. Eng. 10:699. doi: 10.3390/jmse10050699

[ref54] Quince C. Walker A. W. Simpson J. T. Loman N. J. Segata N. (2017). Shotgun metagenomics, from sampling to analysis. Nat. Biotechnol. 35, 833–844. doi: 10.1038/nbt.3935 28898207

[ref55] Ramanan R. Kim B. H. Cho D. H. Oh H. M. Kim H. S. (2016). Algae–bacteria interactions: evolution, ecology and emerging applications. Biotechnol. Adv. 34, 14–29. doi: 10.1016/j.biotechadv.2015.12.003, PMID: 26657897

[ref56] Rambo I. M. Dombrowski N. Constant L. Erdner D. Baker B. J. (2020). Metabolic relationships of uncultured bacteria associated with the microalgae *Gambierdiscus*. Environ. Microbiol. 22, 1764–1783. doi: 10.1111/1462-2920.14878, PMID: 31775181

[ref57] Riemann L. Steward G. F. Azam F. (2000). Dynamics of bacterial community composition and activity during a mesocosm diatom bloom. Appl. Environ. Microbiol. 66, 578–587. doi: 10.1128/AEM.66.2.578-587.2000, PMID: 10653721 PMC91866

[ref58] Sarmento H. Romera Castillo C. Lindh M. Pinhassi J. Sala M. M. Gasol J. M.. (2013). Phytoplankton species-specific release of dissolved free amino acids and their selective consumption by bacteria. Limnol. Oceanogr. 58, 1123–1135. doi: 10.4319/lo.2013.58.3.1123

[ref59] Schapira M. Vincent D. Gentilhomme V. Seuront L. (2008). Temporal patterns of phytoplankton assemblages, size spectra and diversity during the wane of a *Phaeocystis globosa* spring bloom in hydrologically contrasted coastal waters. J. Mar. Biol. Assoc. 88, 649–662. doi: 10.1017/S0025315408001306

[ref60] Seemann T. (2014). Prokka: rapid prokaryotic genome annotation. Bioinformatics 30, 2068–2069. doi: 10.1093/bioinformatics/btu153, PMID: 24642063

[ref61] Segev E. Wyche T. P. Kim K. H. Petersen J. Ellebrandt C. Vlamakis H.. (2016). Dynamic metabolic exchange governs a marine algal-bacterial interaction. elife 5:e17473. doi: 10.7554/eLife.17473, PMID: 27855786 PMC5148602

[ref62] Seymour J. R. Amin S. A. Raina J.-B. Stocker R. (2017). Zooming in on the phycosphere: the ecological interface for phytoplankton–bacteria relationships. Nat. Microbiol. 2:17065. doi: 10.1038/nmicrobiol.2017.65 28555622

[ref63] Seymour J. R. Simó R. Ahmed T. Stocker R. (2010). Chemoattraction to dimethylsulfoniopropionate throughout the marine microbial food web. Science 329, 342–345. doi: 10.1126/science.1188418, PMID: 20647471

[ref64] Shao Q. Lin Z. Zhou C. Zhu P. Yan X. (2020). Succession of bacterioplankton communities over complete *Gymnodinium*-diatom bloom cycles. Sci. Total Environ. 709:135951. doi: 10.1016/j.scitotenv.2019.135951, PMID: 31887501

[ref65] Shemi A. Alcolombri U. Schatz D. Farstey V. Vincent F. Rotkopf R.. (2021). Dimethyl sulfide mediates microbial predator–prey interactions between zooplankton and algae in the ocean. Nat. Microbiol. 6, 1357–1366. doi: 10.1038/s41564-021-00971-3, PMID: 34697459

[ref66] Sunagawa S. Coelho L. P. Chaffron S. Kultima J. R. Labadie K. Salazar G.. (2015). Structure and function of the global ocean microbiome. Science 348:1261359. doi: 10.1126/science.1261359 25999513

[ref67] Teeling H. Fuchs B. M. Becher D. Klockow C. Gardebrecht A. Bennke C. M.. (2012). Substrate-controlled succession of marine bacterioplankton populations induced by a phytoplankton bloom. Science 336, 608–611. doi: 10.1126/science.1218344, PMID: 22556258

[ref68] Teeling H. Fuchs B. M. Bennke C. M. Krüger K. Chafee M. Kappelmann L.. (2016). Recurring patterns in bacterioplankton dynamics during coastal spring algae blooms. elife 5:e11888. doi: 10.7554/eLife.11888, PMID: 27054497 PMC4829426

[ref69] Teng Z. J. Wang P. Chen X. L. Guillonneau R. Li C. Y. Zou S. B.. (2021). Acrylate protects a marine bacterium from grazing by a ciliate predator. Nat. Microbiol. 6, 1351–1356. doi: 10.1038/s41564-021-00981-1, PMID: 34697458

[ref70] Uritskiy G. V. DiRuggiero J. Taylor J. (2018). MetaWRAP—a flexible pipeline for genome-resolved metagenomic data analysis. Microbiome 6:158. doi: 10.1186/s40168-018-0541-1, PMID: 30219103 PMC6138922

[ref71] Verity P. G. Brussaard C. P. Nejstgaard J. C. van Leeuwe M. A. Lancelot C. Medlin L. K. (2007). Current understanding of *Phaeocystis* ecology and biogeochemistry, and perspectives for future research. Biogeochemistry 83, 311–330. doi: 10.1007/s10533-007-9090-6

[ref72] Wemheuer B. Wemheuer F. Hollensteiner J. Meyer F. D. Voget S. Daniel R. (2015). The green impact: bacterioplankton response toward a phytoplankton spring bloom in the southern North Sea assessed by comparative metagenomic and metatranscriptomic approaches. Front. Microbiol. 6:805. doi: 10.3389/fmicb.2015.00805, PMID: 26322028 PMC4531512

[ref73] Wu K. Ying K. Zhou J. Liu D. Liu L. Tao Y.. (2021). Optimizing the growth of *Haematococcus pluvialis* based on a novel microbubble-driven photobioreactor. iScience 24:103461. doi: 10.1016/j.isci.2021.103461, PMID: 34988392 PMC8710528

[ref74] Xu Q. Wang P. Huangleng J. Su H. Chen P. Chen X.. (2022). Co-occurrence of chromophytic phytoplankton and the *vibrio* community during *Phaeocystis globosa* blooms in the Beibu gulf. Sci. Total Environ. 805:150303. doi: 10.1016/j.scitotenv.2021.150303, PMID: 34537702

[ref75] Xu X. Yu Z. He L. Cao X. Chen N. Song X. (2020). Metabolic analyses by metatranscriptomics highlight plasticity in phosphorus acquisition during monospecific and multispecies algal blooms. Hydrobiologia 847, 1071–1085. doi: 10.1007/s10750-019-04169-x

[ref76] Zecher K. Hayes K. R. Philipp B. (2020). Evidence of interdomain ammonium cross-feeding from methylamine- and glycine betaine-degrading *Rhodobacteraceae* to diatoms as a widespread interaction in the marine phycosphere. Front. Microbiol. 11:533894. doi: 10.3389/fmicb.2020.533894, PMID: 33123096 PMC7574528

[ref77] Zhang H. Hou F. Xie W. Wang K. Zhou X. Zhang D.. (2020). Interaction and assembly processes of abundant and rare microbial communities during a diatom bloom process. Environ. Microbiol. 22, 1707–1719. doi: 10.1111/1462-2920.14820, PMID: 31599072

[ref78] Zhang G. Liang S. Shi X. Han X. (2015). Dissolved organic nitrogen bioavailability indicated by amino acids during a diatom to dinoflagellate bloom succession in the Changjiang River estuary and its adjacent shelf. Mar. Chem. 176, 83–95. doi: 10.1016/j.marchem.2015.08.001

[ref79] Zhou J. Lao Y. M. Song J. T. Jin H. Zhu J. M. Cai Z. H. (2020a). Temporal heterogeneity of microbial communities and metabolic activities during a natural algal bloom. Water Res. 183:116020. doi: 10.1016/j.watres.2020.116020, PMID: 32653764

[ref80] Zhou J. Lyu Y. Richlen M. L. Anderson D. M. Cai Z. (2016). Quorum sensing is a language of chemical signals and plays an ecological role in algal-bacterial interactions Crit. Rev. Plant Sci. 35, 81–105. doi: 10.1080/07352689.2016.1172461, PMID: 28966438 PMC5619252

[ref81] Zhou J. Zhang B. Y. Yu K. Du X. P. Zhu J. M. Zeng Y. H.. (2020b). Functional profiles of phycospheric microorganisms during a marine dinoflagellate bloom. Water Res. 173:115554. doi: 10.1016/j.watres.2020.115554, PMID: 32028248

[ref82] Zhu J. Chen G. Zhou J. Zeng Y. Cheng K. Cai Z. (2022). Dynamic patterns of quorum sensing signals in phycospheric microbes during a marine algal bloom. Environ. Res. 212:113443. doi: 10.1016/j.envres.2022.113443, PMID: 35550809

[ref83] Zohdi E. Abbaspour M. (2019). Harmful algal blooms (red tide): a review of causes, impacts and approaches to monitoring and prediction. Int. J. Environ. Sci. Technol. 16, 1789–1806. doi: 10.1007/s13762-018-2108-x

