# Supplementary figures and images for: Microbial community composition and metabolic potential during a succession of algal blooms from Skeletonema sp. to Phaeocystis sp
Source: Front Microbiol. 2023 Apr 17;14:1147187. doi: 10.3389/fmicb.2023.1147187 (PMC10149697; doi:10.3389/fmicb.2023.1147187)

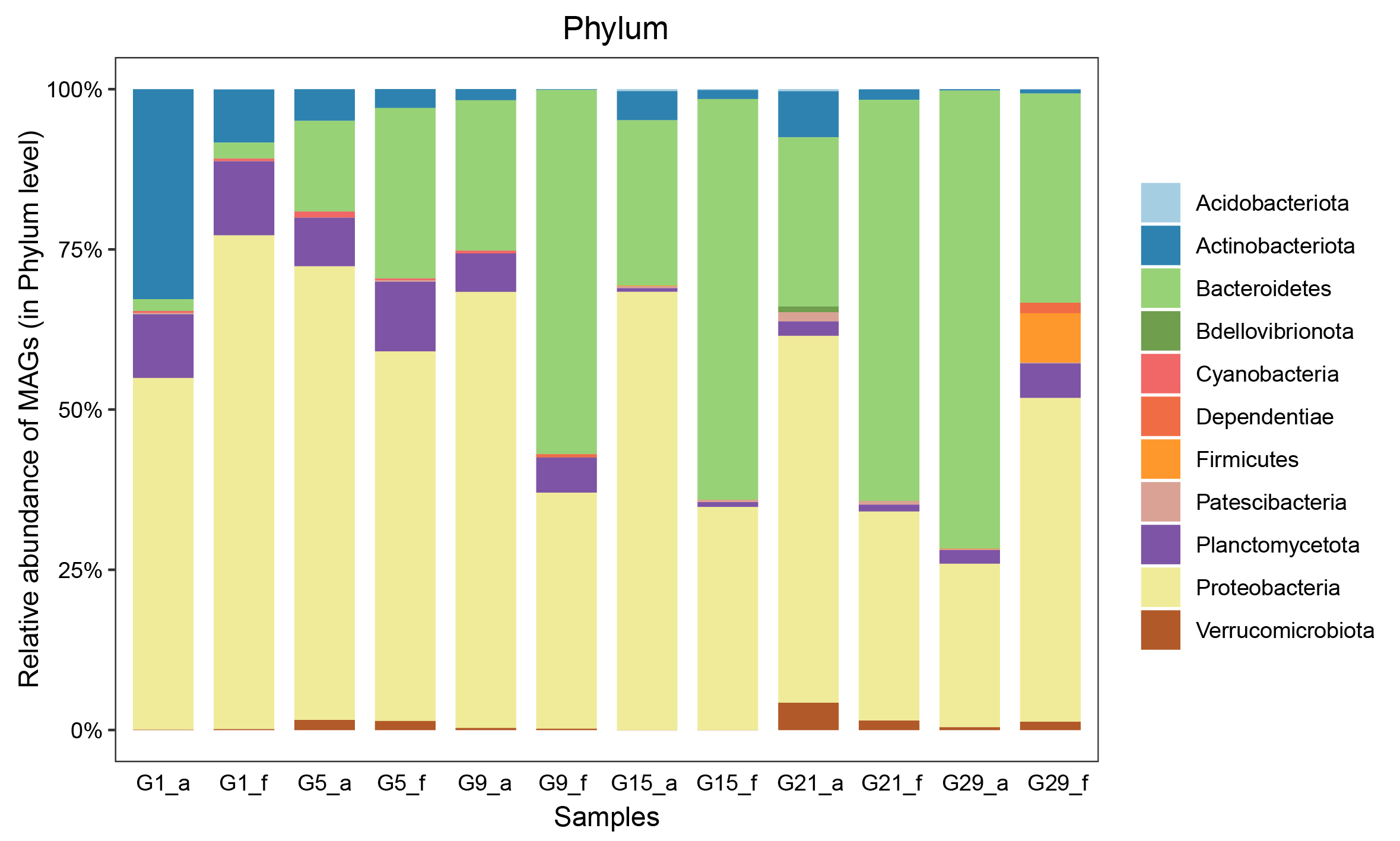

Supplement: Supplementary file 1 [file Image_1.JPEG]

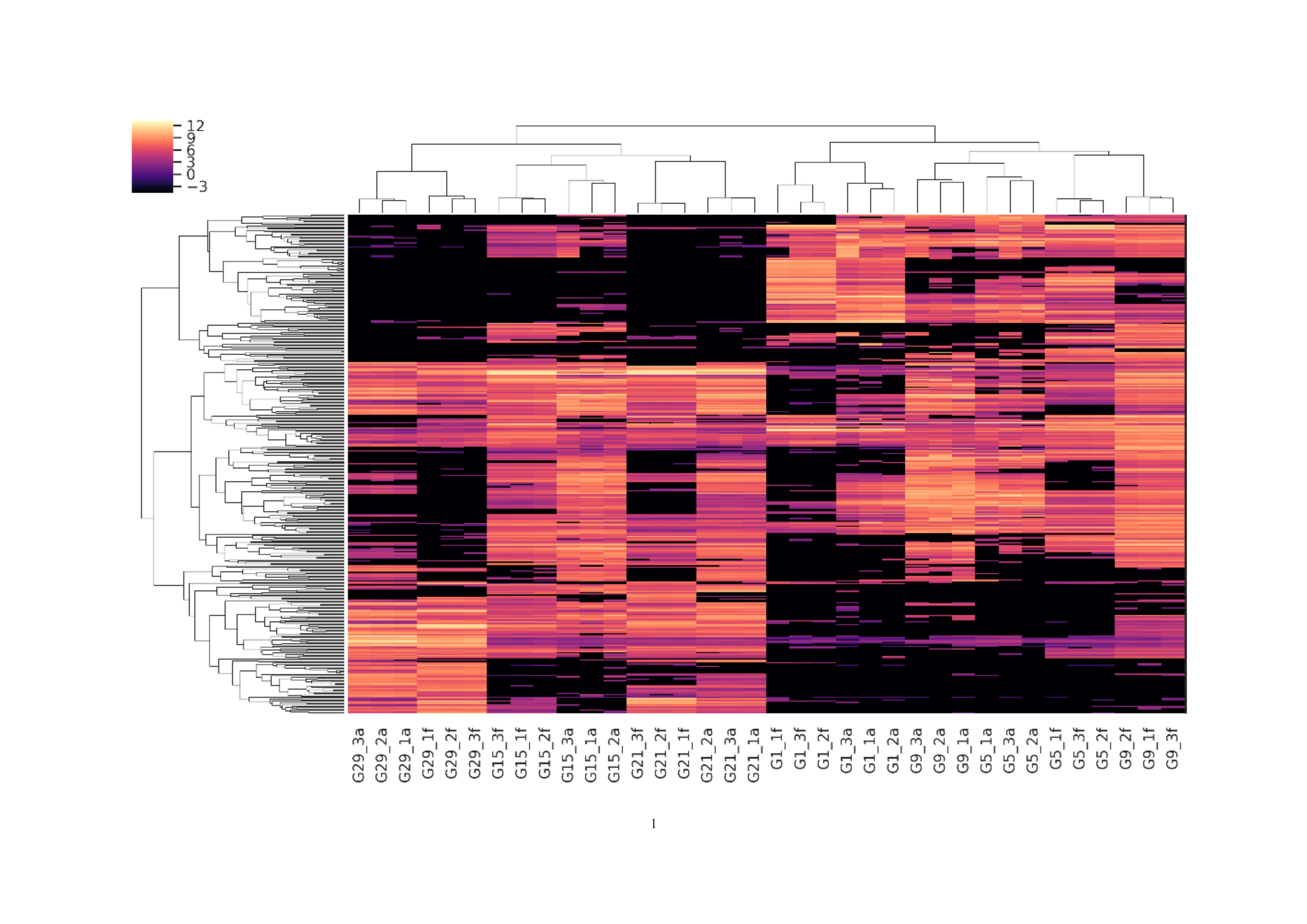

Supplement: Supplementary file 2 [file Image_2.JPEG]
