# Supplementary material for: Microbial community composition and metabolic potential during a succession of algal blooms from Skeletonema sp. to Phaeocystis sp
Source: Front Microbiol. 2023 Apr 17;14:1147187. doi: 10.3389/fmicb.2023.1147187 (PMC10149697; doi:10.3389/fmicb.2023.1147187)
Supplement: Supplementary file 8 [file Data_Sheet_1.docx]

**Supplementary figure legends**

**Table S1.** The classification and relative abundance of all the bins.

**Figure S1.** The bacterial composition in the Phylum level during the succession of blooms.

**Figure S2.** The relative abundances of bacterial MAGs. In the horizontal coordinate, the suffix letter f in the sample name indicates free-living samples, and the suffix letter a indicates attached samples (n = 3). The relative abundance values of MAGs were normalized by Z-score. Average linkage hierarchical clustering algorithm for determining the relationships between MAGs and between samples. The suffixes “a” and “f” denote the attached and free-living groups, respectively.

**Figure S3**. KO absence/presence in MAGs retrieved from the blooms involved in dimethylsulphoniopropionate (DMSP) metabolism, sulfate reduction, and SOX system. Green squares are KOs involved in the same step of the module as defined by KEGG. Branch labels display taxonomy at the lowest inferred level. The heat map shows the relative abundance of bacterial MAGs at each time point again. The row z-score value of each MAGs is plotted on a white-blue color scale. The black bars show the relative abundance of each MAGs in the two bloom stages.

**Figure S4**. KO absence/presence in MAGs retrieved from the blooms involved in vitamin biosynthesis. Green squares are KOs involved in the same step of the module as defined by KEGG. Branch labels display taxonomy at the lowest inferred level. The heat map shows the relative abundance of bacterial MAGs at each time point again. The row z-score value of each MAGs is plotted on a white-blue color scale. The black bars show the relative abundance of each MAGs in the two bloom stages.

**Figure S5**. KO absence/presence in MAGs retrieved from the blooms involved in AI-1 (namely AHLs (acyl-homoserine lactones)), AI-2 (autoinducer-2), DSF (diffusible signal factor), and PQS (quinolone-like2-heptyl-3-hydroxy-4-quinolone). Green squares are KOs involved in the same step of the module as defined by KEGG. Branch labels display taxonomy at the lowest inferred level. The heat map shows the relative abundance of bacterial MAGs at each time point again. The row z-score value of each MAGs is plotted on a white-blue color scale. The black bars show the relative abundance of each MAGs in the two bloom stages.

**Figure S6**. KO absence/presence in MAGs retrieved from the blooms involved in indoleacetic acid (IAA) biosynthesis. Green squares are KOs involved in the same step of the module as defined by KEGG. Branch labels display taxonomy at the lowest inferred level. The heat map shows the relative abundance of bacterial MAGs at each time point again. The row z-score value of each MAGs is plotted on a white-blue color scale. The black bars show the relative abundance of each MAGs in the two bloom stages.
